# Prolonged voluntary wheel running reveals unique adaptations in mdx mice treated with microdystrophin constructs ± the nNOS-binding site

**DOI:** 10.3389/fphys.2023.1166206

**Published:** 2023-06-26

**Authors:** S. E. Hamm, C. Yuan, L. F. McQueen, M. A. Wallace, H. Zhang, A. Arora, A. M. Garafalo, R. P. McMillan, M. W. Lawlor, M. J. Prom, E. M. Ott, J. Yan, A. K. Addington, C. A. Morris, J. P. Gonzalez, R. W. Grange

**Affiliations:** ^1^ Department of Human Nutrition, Foods and Exercise and Metabolism Core, Virginia Tech, Blacksburg, VA, United States; ^2^ Department of Pathology and Neuroscience Research Center, Medical College of Wisconsin and Diverge Translational Science Laboratory, Milwaukee, WI, United States; ^3^ Solid Biosciences, Inc., Cambridge, MA, United States

**Keywords:** endurance, muscle strength, AAV (adeno-associated virus), microdystrophin, longevity

## Abstract

We tested the effects of prolonged voluntary wheel running on the muscle function of mdx mice treated with one of two different microdystrophin constructs. At 7 weeks of age mdx mice were injected with a single dose of AAV9-CK8-microdystrophin with (gene therapy 1, GT1) or without (gene therapy 2, GT2) the nNOS-binding domain and were assigned to one of four gene therapy treated groups: mdxRGT1 (run, GT1), mdxGT1 (no run, GT1), or mdxRGT2 (run,GT2), mdxGT2 (no run, GT2). There were two mdx untreated groups injected with excipient: mdxR (run, no gene therapy) and mdx (no run, no gene therapy). A third no treatment group, Wildtype (WT) received no injection and did not run. mdxRGT1, mdxRGT2 and mdxR performed voluntary wheel running for 52 weeks; WT and remaining mdx groups were cage active. Robust expression of microdystrophin occurred in diaphragm, quadriceps, and heart muscles of all treated mice. Dystrophic muscle pathology was high in diaphragms of non-treated mdx and mdxR mice and improved in all treated groups. Endurance capacity was rescued by both voluntary wheel running and gene therapy alone, but their combination was most beneficial. All treated groups increased *in vivo* plantarflexor torque over both mdx and mdxR mice. mdx and mdxR mice displayed ∼3-fold lower diaphragm force and power compared to WT values. Treated groups demonstrated partial improvements in diaphragm force and power, with mdxRGT2 mice experiencing the greatest improvement at ∼60% of WT values. Evaluation of oxidative red quadriceps fibers revealed the greatest improvements in mitochondrial respiration in mdxRGT1 mice, reaching WT levels. Interestingly, mdxGT2 mice displayed diaphragm mitochondrial respiration values similar to WT but mdxRGT2 animals showed relative decreases compared to the no run group. Collectively, these data demonstrate that either microdystrophin construct combined with voluntary wheel running increased *in vivo* maximal muscle strength, power, and endurance. However, these data also highlighted important differences between the two microdystrophin constructs. GT1, with the nNOS-binding site, improved more markers of exercise-driven adaptations in metabolic enzyme activity of limb muscles, while GT2, without the nNOS-binding site, demonstrated greater protection of diaphragm strength after chronic voluntary endurance exercise but decreased mitochondrial respiration in the context of running.

## Introduction

Duchenne muscular dystrophy (DMD) is a fatal, progressive, muscle wasting disease caused by mutations on the X chromosome that lead to an absence of functional dystrophin. (Yiu and Kornberg, 2015; Duan et al., 2021). Dystrophin is a large muscle protein that links ɣ-actin in the myofiber to the dystrophin-glycoprotein complex (DGC), which spans the sarcolemma. (Gao and McNally, 2015; Duan et al., 2021). The DGC thus links the inside of the muscle fiber to the extracellular matrix and provides several important signaling functions for the cell. (Gao and McNally, 2015). Absence of dystrophin leads to loss of the DGC and all of its functions. (Gao and McNally, 2015; Duan et al., 2021). Consequently, muscles are susceptible to damage and necrosis, resulting in loss of ambulation, cardiomyopathy, and respiratory failure. (Gao and McNally, 2015; Duan et al., 2021).

Treatment for patients with DMD has historically been corticosteroids, which reduce inflammation and slow disease progression. (Duan et al., 2021). Recently, gene-based therapeutics such as induction of utrophin overexpression, exon-skipping drugs, and microdystrophin gene therapy have been tested in clinical trials. (Duan et al., 2021). Microdystrophin gene therapy delivers a truncated version of dystrophin, engineered to contain the most important parts of dystrophin to retain function, but small enough to fit into the available adeno-associated viral (AAV) vectors (∼5 kb). (Chamberlain and Chamberlain, 2017; Duan, 2018a). Two important microdystrophin constructs include H2µDys and µDys-5R. Versions of each are being used in clinical trials. (Duan, 2018a; Duan, 2018b; Ramos et al., 2019). The structure of each contain an actin-binding domain (ABD), Hinge domain 1 (H1) and 4 or 5 spectrin-like repeats (R). (Duan, 2018b). Both constructs also contain Hinge domain 4 (H4) and a portion of the cysteine-rich region (CR) that is critical for binding to β-dystroglycan in the DGC. (Duan, 2018b). H2µDys includes Hinge domain 2, which contains a polyproline site proposed to be detrimental to structure of the cytoskeleton and extracellular matrix when delivered to mdx mice. (Banks et al., 2010). µDys-5R is known for its unique nNOS-binding site at R16 and R17, which has been shown to restore nNOS localization to the sarcolemma and reduce functional ischemia in the limbs during exercise and, independent of exercise, lead to increased force output over other microdystrophins. (Lai et al., 2009; Lai et al., 2013; Zhang et al., 2013; Ramos et al., 2019). With potential success of clinical trials forthcoming, it is becoming increasingly important to determine the limitations of these microdystrophin constructs, especially in relation to exercise. (Harper et al., 2002; Liu et al., 2005; Bostick et al., 2011).

Voluntary wheel running is beneficial to mdx muscle, (Dupont-Versteegden et al., 1994; Landisch et al., 2008; Baltgalvis et al., 2012; Selsby et al., 2013), whereas other exercise modalities such as forced treadmill training regimens exacerbate the dystrophic condition. (Aartsma-Rus and van Putten, 2014; Schill et al., 2016; Rodgers et al., 2020). Few studies have examined the effects of exercise on mini- or microdystrophin gene therapy. (Shin et al., 2011; Zhang et al., 2013; Rodgers et al., 2020; Hamm et al., 2021). When forced treadmill running was used, studies report at least partial rescue of muscle function from exercise-induced damage. (Shin et al., 2011; Rodgers et al., 2020). mdx mice treated with microdystrophin that performed voluntary wheel running for 13 days ran farther than their untreated controls, but other aspects of skeletal muscle function were not measured. (Shin et al., 2011). In a recent study, we demonstrated that 21 weeks of voluntary wheel running was beneficial to young mdx mice treated with microdystrophin gene therapy. (Hamm et al., 2021).

The objectives of our current study were to determine if (1) prolonged (52 weeks) voluntary wheel running and (2) microdystrophin construct structure (± nNOS binding site) in mdx mice will sustain the functional benefits we reported for microdystrophin combined with short-term (21 weeks) voluntary wheel running in mdx mice (Hamm et al., 2021). To address these two objectives, we assessed the effects of 52 weeks of voluntary wheel running on whole body endurance and muscle function in young mdx mice treated with µDys-5R (with the nNOS-binding site, gene therapy 1, GT1) or H2µDys (without the nNOS-binding site, gene therapy 2, GT2) microdystrophin gene therapy constructs. Our data demonstrate that either the GT1 or the GT2 gene therapy improved several functional assessments similarly in mdx mice, with or without voluntary wheel running. Treated mice with either the GT1 or the GT2 microdystrophin construct and did not run displayed enhanced endurance capacity (i.e., time to exhaustion on a treadmill running test) and improved plantarflexor torque. Mice that were treated with either the GT1 or the GT2 microdystrophin construct and performed voluntary wheel running exhibited further improvement in endurance capacity and similar improvements in plantarflexor torque. However, improvements in *ex vivo* contractile assessments and metabolic assays were dependent on the GT1 or GT2 microdystrophin construct and activity level (i.e., run versus no run). Thus, prolonged voluntary wheel running complemented the functional improvements provided by both microdystrophin constructs but also revealed the unique benefits of each microdystrophin construct to dystrophic muscle.

## Materials and methods

### Animal studies

**FIGURE 1:**
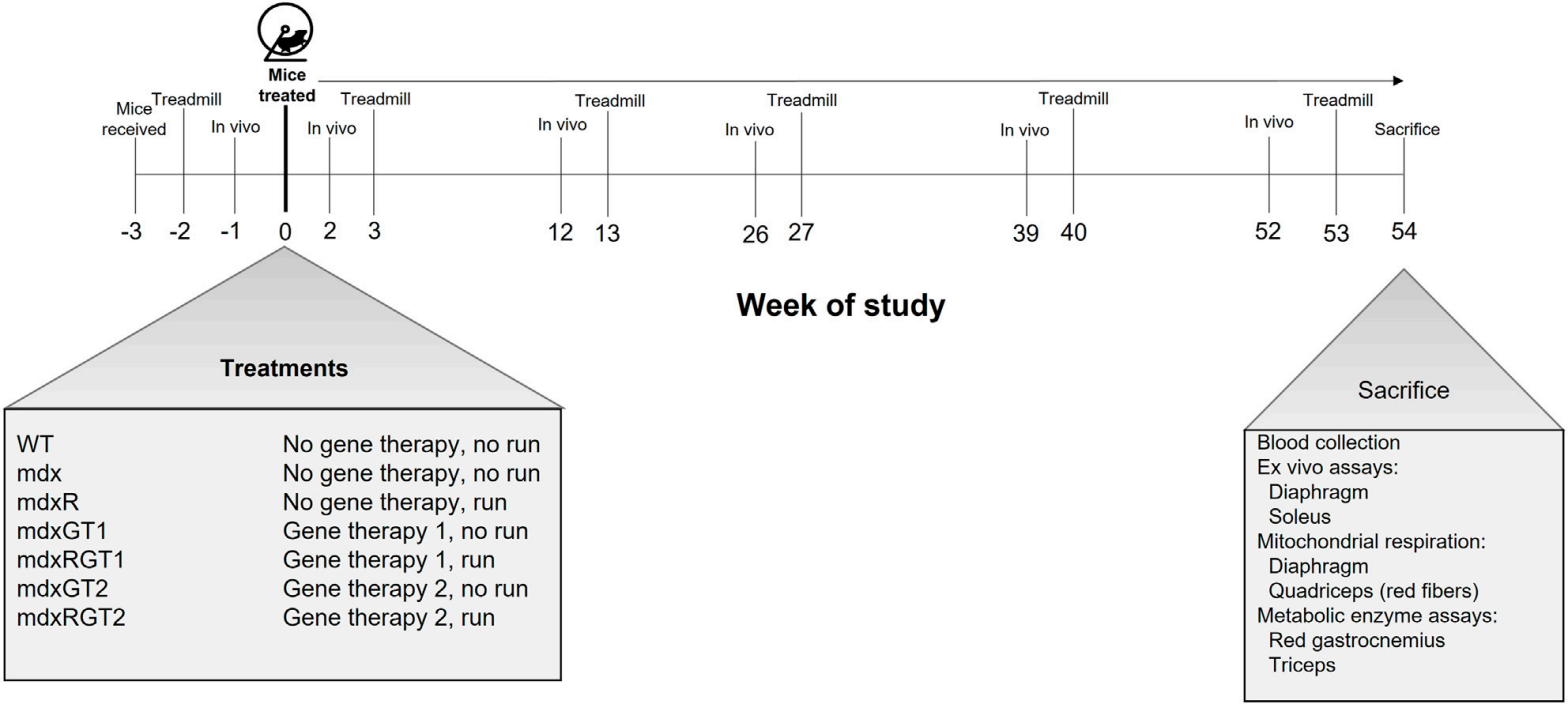
Timeline of study and mouse groups. Mice were received at age 4 weeks (Week−3) and administered treatment and housed with wheels at age 7 weeks (Week 0). mdxR, mdxRGT1, and mdxRGT2 groups had access to a running wheel from Week 0 through 54. Mice were assessed for *in vivo* plantarflexor torque at Weeks -1, 2, 12, 39 and 52. Treadmill runs to exhaustion were conducted at Weeks -2, 3, 13, 27, 40 and 53. Mice were sacrificed and various assays conducted at Age 61 weeks (Week 54). At start of study, *n* = 8 each group. See Materials and methods for details.

All animal experiments were approved by the Institutional Animal Care and Use Committee at Virginia Tech, and in concordance with NIH guidelines. Four-week-old male wildtype (WT; C57BL-10ScSnJ/Jax strain #000476) and male mdx (C57BL/10ScSn-DMDmdx/Jax strain #001801) mice were purchased from the Jackson Laboratory (Bar Harbor, ME; WT *n* = 8, mdx *n* = 48). The total of 56 mice were purchased in four cohorts of 10 and two cohorts of 8 mice (age 4 weeks) over 6 weeks. Mice in each cohort were assigned to one of the experimental groups each week (e.g., 1-2 mice/experimental group). Mice in both WT and the various mdx groups were staggered over the 6 weeks, e.g., not all WT mice started the same week, but were distributed over the 6 weeks. The mdx groups were similarly distributed. All groups (n = 8) followed the same timeline (Figure 1). It was necessary to stagger the groups so assays post-sacrifice could also be staggered over 6 weeks at the end of the study, i.e., data could not be collected from 56 mice if they all started the study simultaneously. Mice were initially group-housed (3-4 mice/cage) in a temperature-controlled room (21.1°C) with a 12-h light/12-h dark cycle and were given access to water and chow (Harlan-Teklad 2018) *ad libitum* until 7 weeks of age. The environmental and water/food conditions were maintained throughout the study. At 7 weeks of age, mice were treated with gene therapy (treated groups; mdxGT1, mdxRGT1, mdxGT2, mdxRGT2) or excipient (non-treated groups; WT, mdx, mdxR) and housed with free-spinning wheels (running groups; mdxR, mdxRGT1, mdxRGT2) or locked wheels (cage-active groups; WT, mdx, mdxGT1, mdxGT2). N values were variable during the study due to various uncontrollable complications, primarily the unexpected illness and/or death of mice (Supplementary Table S8). Mouse masses at sacrifice are reported in Supplementary Table S9.

### Voluntary wheel running

Protocol was followed as previously described. (Hamm et al., 2021). All mice were single housed starting at age 7 weeks (Week 0, Figure 1) after appropriate excipient or microdystrophin injection. Mice in mdxR, mdxRGT1, and mdxRGT2 groups were single housed in cages with running wheels. Mice in WT, mdx, mdxGT1, and mdxGT2 groups were single housed in cages with locked running wheels. All running mice ran 54 weeks. However, the mice were sacrificed on different days (i.e., Monday vs. Friday) during week 54, so mice ran until sacrifice at 54 weeks, but we reported running distance for 52 weeks to standardize the run duration between mice. Thus, our wheel running figure (Figures 5D, E) only shows distances for 0–52 weeks.

### AAV construct and delivery

Two microdystrophin constructs were tested. Recombinant AAV9 was produced at the Vector Core of the University of Massachusetts Medical School. Both constructs were under the control of the creatine kinase 8 (CK8) skeletal and cardiac muscle-specific promoter. The gene-of-interest (GOI) plasmid used for production of the GT1 construct coded for a five-repeat microdystrophin sequence, μDys-5R, which contains the R16/R17 nNOS-binding specific repeats. The gene-of-interest (GOI) plasmid used for production of the GT2 construct coded for a four-repeat microdystrophin sequence, H2μDys, which does contain the R16/R17 nNOS-binding specific repeats. Animals were administered a single dose of each construct at a dose of 2 × 10^14^ vg/kg or an equivalent volume of excipient (phosphate buffered saline + 0.001% Pluronic F-68) via tail vein injection at age 7 weeks with a U100 28Gx1/2 insulin syringe (Becton Dickson, Franklin Lakes, *NJ*).

### Assessment of microdystrophin protein by western blot and immunofluorescence

#### Western blot

Total protein from diaphragm, heart, and quadriceps collected at the conclusion of the study was prepared to determine the content of microdystrophin as previously described (Hamm et al., 2021). Microdystrophin protein levels were compared to a standard microdystrophin reference sample characterized as 100% of normal dystrophin and run on each blot alongside study samples to allow for blot-to-blot interpretation. Relative protein levels were evaluated using a one-way ANOVA with Prism software (GraphPad Prism 9.0).

#### Immunofluorescence

Isopentane-frozen diaphragm strip, heart, and quadriceps muscle specimens obtained at the conclusion of the study were sectioned at 8-μm thickness and immunostained per standard techniques for dystrophin/microdystrophin (Leica, NCL-DYSB) as previously described. (Hamm et al., 2021). Evaluation of percent dystrophin- or microdystrophin-positive fibers was performed by a neuropathologist using a standard fluorescent microscope and estimated to the nearest 5%.

#### Assessment of dystrophic pathology

H&E-stained sections of frozen diaphragm, quadriceps, and heart muscle tissues collected at the conclusion of the study were assessed for histological features specific to myofiber degeneration and regeneration associated with dystrophic muscle pathology as previously described. (Hamm et al., 2021). Dystrophic grade was assigned by the estimated proportion of tissue that showed evidence of active/recent dystrophic changes, including myonecrosis, inflammation, myophagocytosis, and basophilic fibers. All of these pathological findings correspond to evidence of recent muscle damage that would have occurred during the post-treatment period. More chronic dystrophic changes (internal nucleation, fibrosis, fatty replacement) are not an element of this dystrophic grading system, as it is not possible to determine whether these changes were present before treatment was started. The grading scale used for reporting is as follows: Grade 0 = normal, grade 1 = chronic regenerative changes only, grade 1.5 = very mild (<5% of muscle area with active dystrophic pathology), grade 2 = mild (6%–20% of muscle area with active dystrophic pathology), grade 2.5 = mild to moderate (21%–30% of muscle area with active dystrophic pathology), grade 3 = moderate (31%–50% of muscle area with active dystrophic pathology), grade 3.5 = moderate to severe (51%–60% of muscle area with active dystrophic pathology) and grade 4 = severe (>60% of muscle area with active dystrophic pathology).

### Assessments before, during, and after the period of voluntary wheel running

#### Treadmill fatigue tests

A treadmill fatigue test was used to determine endurance capacity. Endurance capacity is defined as the ability of the mouse to exercise for an extended period of time as demonstrated by time to exhaustion during a treadmill running test. All mice were subjected to treadmill training and fatigue tests as previously described. (Hamm et al., 2021). Briefly, mice were subjected to a short training protocol on a 6-lane treadmill (Columbus Instruments) for 3 days and a fatigue test on the fourth day. For the fatigue test, there is a progressive increase in speed every 2 min for 5 steps starting at 0.02 to 0.1 m/s, in 0.02 m/s increments and an increase of 0.1 m/s on the sixth step to 0.2 m/s. For steps 7–11, speed is increased every 1,200 s from 0.3 to 0.5 m/s in 0.05 m/s increments. The test is over when the mouse is fatigued after three failures. Failure is defined as the inability of the mouse to keep running after aggressive physical nudges with a bottle brush (Fisher). Each mouse was allowed three failures before it was considered fatigued. The treadmill was paused using a custom pause function in the instrument software and time to fatigue was recorded. Fatigued mice were quickly removed from the treadmill and the protocol resumed promptly for the remaining mice. Fatigue tests were performed at Baseline (2 weeks pre-treatment) and subsequently following each *in vivo* contractile assessment (3, 13-, 27-, 40-, and 53-weeks post-treatment; Figure 1).

#### *In vivo* skeletal muscle contractile properties

*In vivo* isometric plantarflexor torque and torque-velocity were assessed 1 week prior to, and at 2-, 12-, 26-, 39-, and 52-weeks after treatment to determine plantarflexor contractile performance (Figure 1). Body mass was recorded prior to each experiment. Mice were anesthetized with isoflurane (VetOne Fluriso, Boise, Idaho) and prepared as previously described. (Hamm et al., 2021). Following the experiment, mice recovered in a clean cage on top of a heated pad. After mice were fully awake and mobile in the recovery chamber, they were placed in a clean cage with an active or locked running wheel as necessary and returned to the vivarium. *In vivo* contractile properties were determined using methods described previously. (Hamm et al., 2021).

Isometric torque-frequency experiments were performed as described. (Hamm et al., 2021). Briefly, the plantarflexors were stimulated in a series of increasing frequencies from 1–200 Hz. Data were plotted as torque normalized to mouse body mass (mN x m/g) vs. frequency.

Torque-velocity was determined by measuring torque output at set velocities. (Baltgalvis et al., 2012). In this method, the pedal moves passively to 19° dorsiflexion, then the plantarflexors are stimulated at 300 Hz while the pedal moves to 19° plantarflexion at a set velocity. Torque was measured at 1,200°/s, 1,000°/s, 800°/s, 600°/s, 400°/s, 200°/s, and 100°/s, with stimulation durations of 0.0317, 0.038, 0.0475, 0.063, 0.095, 0.190, 0.380s, respectively. Due to the high stimulation frequency, 3 min of rest were given between each stimulation. Peak torque during active stimulation was determined. Peak torque was multiplied by angular velocity to obtain power values. Values were normalized to body mass (g).

### Assessments of isolated soleus and diaphragm muscle contractile properties

#### *Ex vivo* contractile properties

Soleus muscles and diaphragm strips were carefully dissected and placed in a jacketed water bath to determine contractile properties including force, velocity, power, and responses to eccentric contractions as previously described. (Hamm et al., 2021).

#### Assessments of mitochondrial respiration

Permeabilized fibers of red (oxidative) quadriceps were prepared and high resolution O_2_ consumption measurements were performed as previously described to determine mitochondrial respiration. (Hamm et al., 2021). Similarly, diaphragm fibers were permeabilized with a saponin treatment, and fibers were washed and placed in buffer in the chamber of an Oroboros Oxygraph-2k (Oroboros Instruments, Innsbruck, Austria) as described. O_2_ consumption was measured using a series of substrate injections to elicit activity of mitochondrial enzymes of the electron transport chain similar to the procedure used for quadriceps fibers, with a few key differences. Substrate injections were as follows: 10 mM glutamate/malate, 0.5uM rotenone, 10 mM succinate, 5 mM ADP, and 0.5 mM FCCP. Fiber bundles were removed from the chamber, washed with dH_2_O, and freeze dried as previously described. Freeze-dried bundles were weighed, and data were normalized to mass (mg) of the bundles.

#### Assessment of metabolic enzyme activities

Red and white gastrocnemius, as well as whole triceps, were prepared and assayed as previously described to determine the activities of various metabolic enzymes. (Hamm et al., 2021). Citrate Synthase (CS), Malate Dehydrogenase (MD), and Cytochrome c oxidase (COX) were assayed in red and white gastrocnemius and whole triceps. Phosphofructokinase (PFK) and β-hydroxyacyl - CoA dehydrogenase (βHAD)were assayed in red gastrocnemius and whole triceps.

### Statistical analysis

GraphPad Prism 9.0 was used to perform all statistical analyses. Data were analyzed with a one-way (group) or a two-way ANOVA (e.g., group x time) as required. Treatment group was considered a single independent variable, instead of gene therapy or running each as independent variables. Thus, a one-way ANOVA was used for simple comparisons like microdystrophin content (independent variable: group, dependent variable: microdystrophin content) or treadmill time to fatigue (independent variable: group, dependent variable: time to fatigue). A two-way ANOVA was used when we tested the effects of two independent variables such as group and time on torque output (independent variable1: group, independent variable 2: time, dependent variable: torque). If a significant interaction between two factors occurred, Tukey’s HSD test was used to determine differences between means. Data are presented as mean ± SEM. Statistical significance was accepted at *p* < 0.05.

## Results

### Study strategy

In this study, we aimed to determine if µDys-5R (GT1, with the nNOS binding site) and H2µDys (GT2, without the nNOS binding site) treatment would improve running performance and muscle function in mdx mice during prolonged voluntary wheel running for 52-weeks (Figure 1). Treated animals were separated into gene therapy (mdxGT1, *n* = 8; mdxGT2, *n* = 8) and gene therapy combined with voluntary wheel running (mdxRGT1, *n* = 8; mdxRGT2, *n* = 8) groups. Each treated group was administered a single intravenous dose of AAV9-CK8-microdystrophin at approximately 7 weeks of age and followed for 54 weeks post treatment. Control groups of WT sedentary mice (*n* = 8), mdx sedentary mice (*n* = 8), and mdx mice that performed voluntary wheel running (mdxR, *n* = 8) were evaluated alongside treated groups throughout the study. *In vivo* plantarflexor contractile assessments and treadmill fatigue tests were performed to test plantarflexor and whole-body muscle function, respectively. At the end of the study, tissues were collected for analysis of microdystrophin protein expression, dystrophic muscle pathology, and mitochondrial respiration. Diaphragm strips and whole soleus muscles were isolated for *ex vivo* contractile analyses. The n values for the majority of assessments after baseline measures and during and after completion of the study were WT, 7; mdx, 6; mdxR, 5; mdxGT1, 7; mdxRGT1, 8; mdxGT2, 7; mdxRGT2, 7; specific n values for each assessment are reported in Supplementary Table S8.

### Diaphragm, quadriceps, and heart muscles displayed robust expression of microdystrophin protein, demonstrated by both Western blot and quantification of immunofluorescence

#### Western blot

**FIGURE 2:**
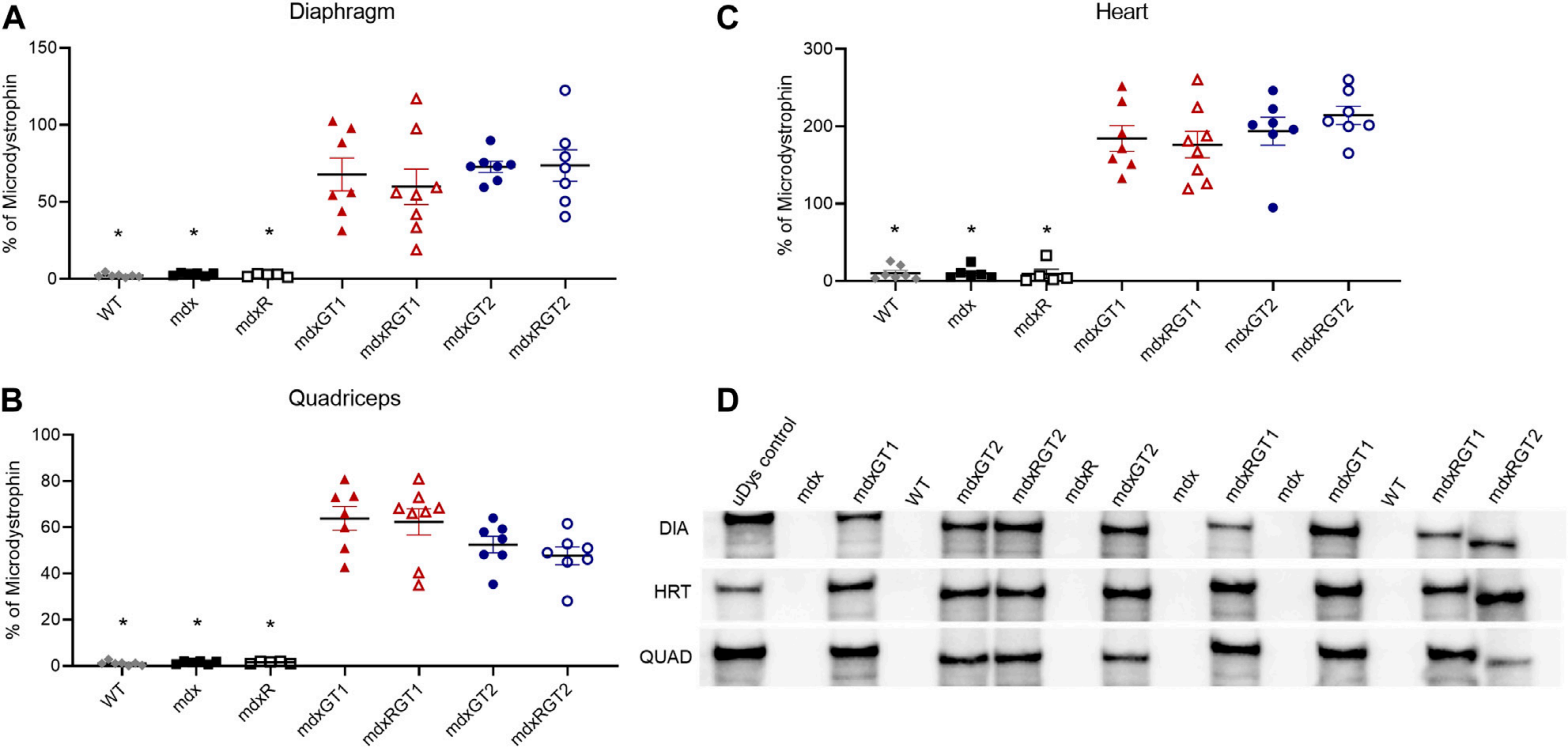
Microdystrophin protein content in diaphragm, quadriceps, and heart by quantification of volume of microdystrophin bands on western blot **(A–C)**. (A)* WT, mdx, mdxR < mdxGT1, mdxRGT1, mdxGT2, mdxRGT2. (B)* WT, mdx, mdxR < mdxGT1, mdxRGT1, mdxGT2, mdxRGT2. (C)* WT, mdx, mdxR < mdxGT1, mdxRGT1, mdxGT2, mdxRGT2. **(D)** Representative western blots of uDys-5R (GT1; 147 kDa) and H2μDys (GT2; 138 kDa) microdystrophin constructs. All comparisons *p* < 0.05.

**FIGURE 3:**
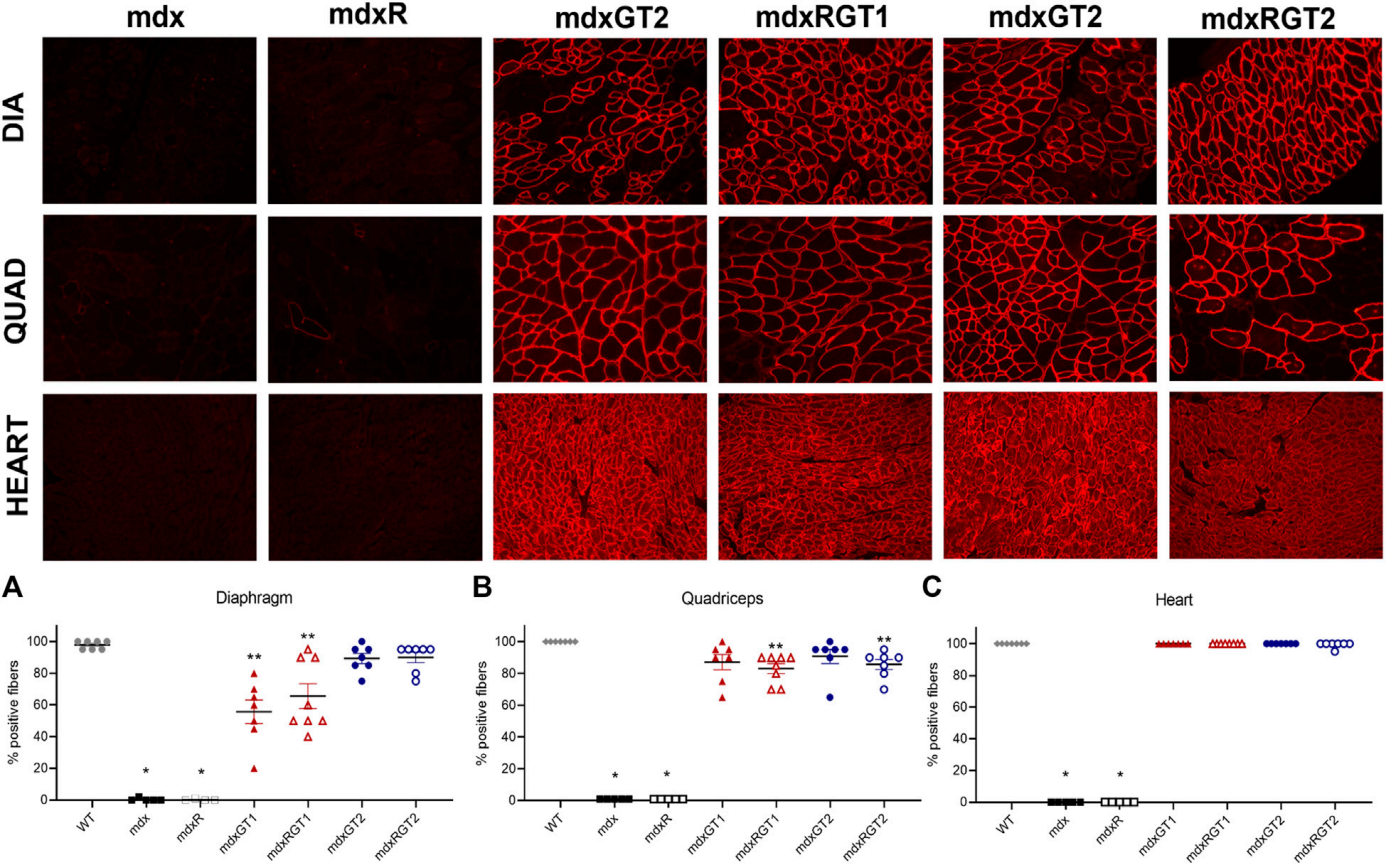
Microdystrophin protein content in diaphragm, quadriceps, and heart by quantification of immunofluorescence **(A–C)**. (A)*mdx, mdxR < all groups. **mdxGT1, mdxRGT1 < WT, mdxGT2, mdxRGT2. (B)* mdx, mdxR < all groups. **mdxRGT1, mdxRGT2 < WT. (C)* mdx, mdxR < all groups. Representative images of diaphragm (DIA), quadriceps (QUAD), and heart from treated groups. All comparisons *p* < 0.05.

Microdystrophin protein levels were evaluated via Western blot (Figure 2; Supplementary Table S1) and immunofluorescence (Figure 3; Supplementary Table S2) to assess expression in muscles. Western blot data for each sample were normalized to a standard microdystrophin reference sample characterized as 100% of normal dystrophin that was run alongside study samples on each blot and expressed as mean % ± SEM. WT (2 ± 0), mdx (3 ± 0) and mdxR (2 ± 0) diaphragm samples were negative for microdystrophin protein bands at 147 kDa (Figure 2D) and quantified at relative % of microdystrophin levels similar to background. Microdystrophin levels were significantly increased to levels of 73 ± 4 for mdxGT2 and 74 ± 10 for mdxRGT2 mice (*p* < 0.05). mdxGT1 and mdxRGT1 mice displayed similarly strong relative expression at 68 ± 11 and 60 ± 12, respectively (**Figure 2A**; *p* < 0.05). Quadriceps from treated mice displayed increased relative expression across groups compared to the background levels in WT (1 ± 0), mdx (1 ± 0), and mdxR (2 ± 0) untreated control samples (**Figure 2B**; mdxGT1: 64 ± 5; mdxRGT1: 62 ± 6; mdxGT2: 53 ± 4; mdxRGT2: 48 ± 4; *p* < 0.05). Robust microdystrophin expression was observed in heart muscle of treated animals regardless of construct compared to background levels in WT (10 ± 3), mdx (10 ± 3), and mdxR (9 ± 6) samples (Figure 2C; mdxGT1: 184 ± 17; mdxRGT1: 176 ± 17; mdxGT2: 194 ± 18; mdxRGT2: 214 ± 12; *p* < 0.05).

#### Immunofluorescence

Immunofluorescence analysis was performed to evaluate the number of fibers positive for anti-dystrophin antibody (DYSB) staining to detect the presence of full-length dystrophin or microdystrophin and expressed as mean % ± SEM. Compared to the mdx or mdxR revertant positive fiber levels (0–1) in diaphragm, quadriceps, and heart, all treated groups demonstrated greater expression (Figure 3; Supplementary Table S2; *p* < 0.05). Higher microdystrophin expression was evident in GT2 treated diaphragms (mdxGT2 89 ± 3; mdxRGT2 90 ± 3) compared to GT1 (mdxGT1 56 ± 7; mdxRGT1 66 ± 8; *p* < 0.05), regardless of activity level (Figure 3A). Analysis of quadriceps showed treated mice had similar levels of microdystrophin positive fibers (Figure 3B; mdxGT1 87 ± 5; mdxRGT1 83 ± 3; mdxGT2 91 ± 4; mdxRGT2 86 ± 3). However, mdxRGT1 and mdxRGT2 showed a running-dependent decrease in microdystrophin compared to WT levels of dystrophin (100 ± 0; *p* < 0.05). Robust expression of microdystrophin was confirmed in heart muscle of all treated groups (mdxGT1 100 ± 0; mdxRGT1 100 ± 0; mdxGT2 100 ± 0; mdxRGT2 99 ± 1), which were rescued to WT levels of dystrophin (Figure 3C; 100 ± 0). Representative immunofluorescence images are shown for each treated mdx group (Figure 3).

### Both microdystrophin constructs similarly improved dystrophic muscle pathology in animals performing voluntary exercise

**FIGURE 4:**
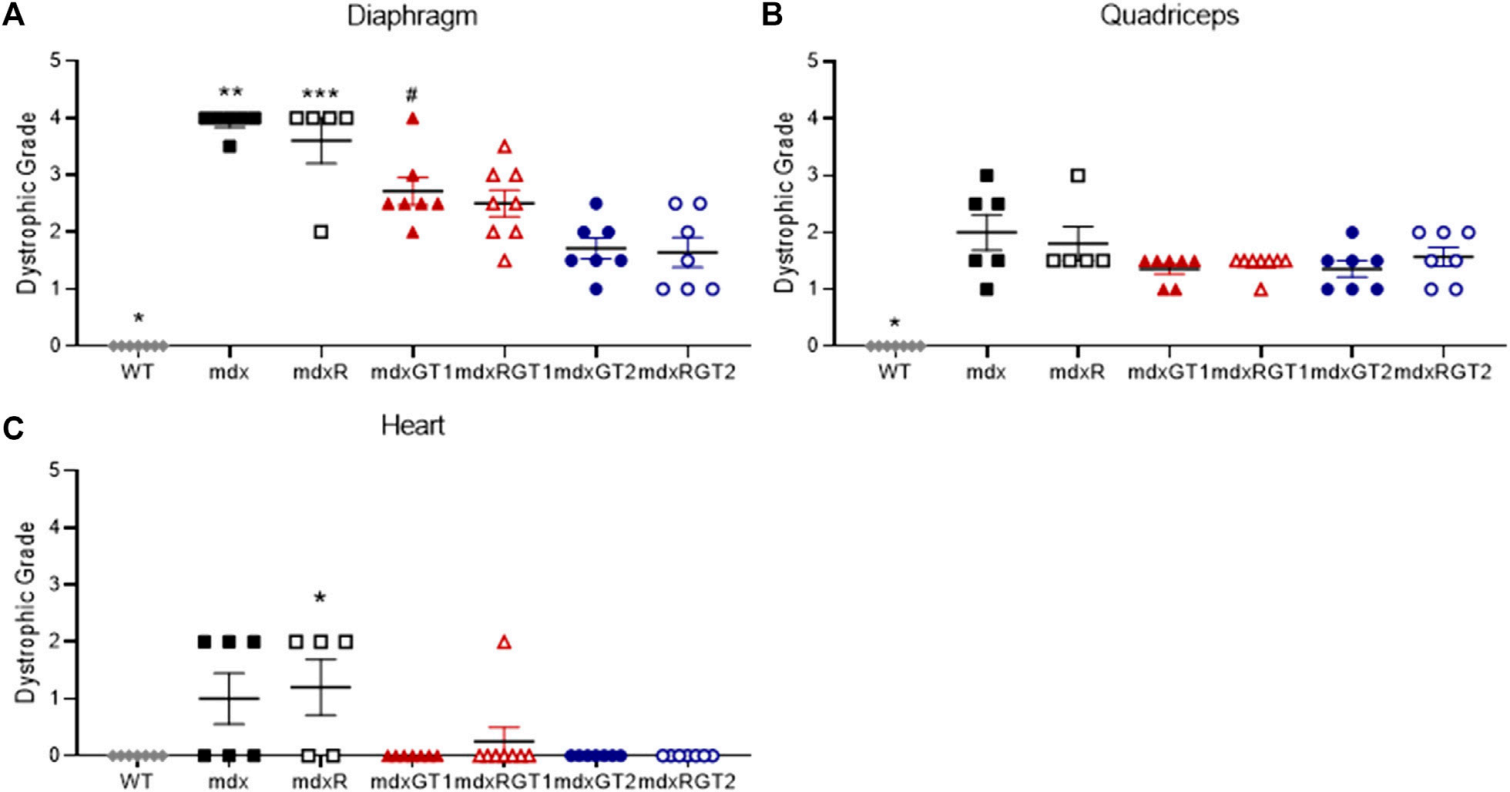
Dystrophic muscle pathology of diaphragm, quadriceps, and heart presented as dystrophic grade. **(A)** Diaphragm *WT < all groups. **mdx > all groups except mdxR. ***mdxR > WT, mdxRGT1, mdxGT2, mdxRGT2. ^#^mdxGT1 >mdxGT2, mdxRGT2. **(B)** Quadriceps *WT < all groups. **(C)** Heart *mdxR < WT, mdxGT1, mdxGT2, mdxRGT2. All comparisons *p* < 0.05.

Dystrophic muscle pathology in the diaphragm, heart, and quadriceps was assessed at the end of the study (Figure 4; Supplementary Table S3). Representative images of the diaphragm are shown for each mdx group (Supplementary Figure S1). Data are presented as mean ± SEM. Diaphragm dystrophic pathology was high in mdx (3.9 ± 0.1) and mdxR (3.6 ± 0.4) mice, but improved in mdxGT1 (2.7 ± 0.2), mdxRGT1 (2.5 ± 0.2), mdxGT2 (1.7 ± 0.2), and mdxRGT2 (1.6 ± 0.3) groups (all *p* < 0.05). Dystrophic pathology was low in heart muscle of all groups (WT 0.0 ± 0.0; mdx 1.0 ± 0.4; mdxR 1.2 ± 0.5; mdxGT1 0.0 ± 0.0; mdxRGT1 0.3 ± 0.3; mdxGT2 0.0 ± 0.0; mdxRGT2 0.0 ± 0.0). No differences in quadriceps dystrophic pathology were observed between treated and untreated mice, regardless of construct or activity level (WT 0.0 ± 0.0; mdx 2.0 ± 0.3; mdxR 1.8 ± 0.3; mdxGT1 1.4 ± 0.1; mdxRGT1 1.4 ± 0.1; mdxGT2 1.4 ± 0.1; mdxRGT2 1.6 ± 0.2).

### Both microdystrophin gene constructs improved time to fatigue, but voluntary wheel running further enhanced endurance capacity

**FIGURE 5:**
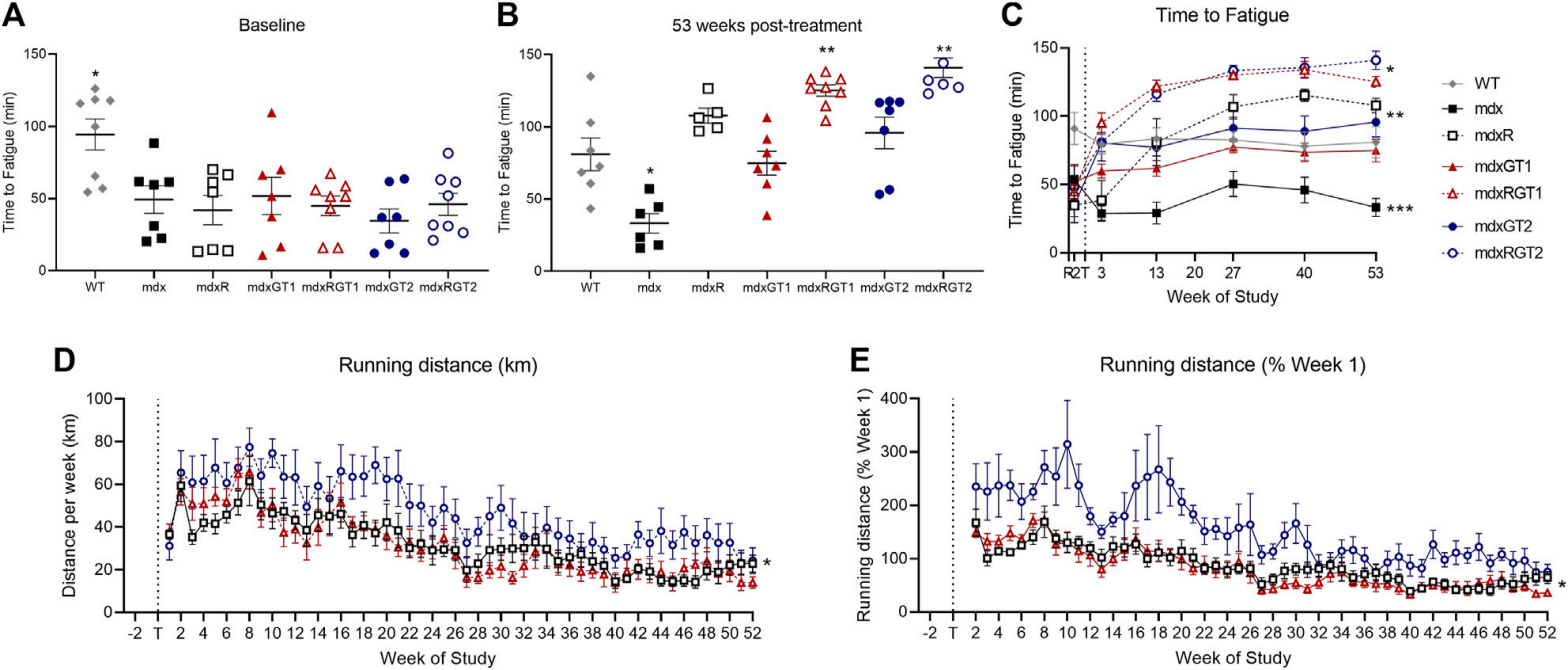
Running capacity. Treadmill fatigue test **(A–C)** and running wheel activity **(D, E)**. **(A)** Baseline treadmill time to fatigue. *WT > all groups. **(B)** 53 weeks post-treatment treadmill time to fatigue. *mdx < all groups. **mdxRGT1, mdxRGT2 > WT, mdxGT1, mdxGT2. **(C)** Treadmill time to fatigue at all timepoints. *mdxRGT1, mdxRGT2 > all groups post-treatment. *mdxR, mdxGT2 > mdxGT1 post-treatment. ***mdx < all groups post-treatment. **(D)** Running wheel distance per week. *mdxRGT2 > mdxR, mdxRGT1. **(E)** Running wheel activity as percent of week 1. *mdxRGT2 > mdxR, mdxRGT1. All comparisons *p* < 0.05.

Treadmill fatigue tests were performed to assess whole-body muscle function (Figure 5; Supplementary Table S4). Time to fatigue data are presented as mean ± SEM minutes. Baseline (2 weeks before treatment) and Final (53 weeks post-treatment) treadmill fatigue tests demonstrate depressed running ability in mdx mice (Baseline 54 ± 10; Final 33 ± 7 min) by 50% compared to WT (Figures 5A–C; Baseline 91 ± 12; Final 81 ± 11 min; *p* < 0.05). In the final test of the study, running ability was rescued by both running alone (mdxR 108 ± 5 min, *p* < 0.05) and gene therapy alone (Figure 5B; mdxGT1 75 ± 8 min; mdxGT2 96 ± 11 min; *p* < 0.05). Mice treated with gene therapy combined with voluntary wheel running (mdxRGT1 125 ± 4 min; mdxRGT2 141 ± 7 min; *p* < 0.05) experienced the greatest benefit, regardless of gene therapy construct (Figure 5B). Interestingly, mdxR mice improved treadmill time ∼46% by week 13 (81 ± 17 min) and by week 53, times were similar to mdxGT2 mice (Figures 5B,C). Voluntary wheel running distance was recorded over 52 weeks (Figures 5D,E; Supplementary Table S5). Weekly running distance is presented as mean ± SEM kilometers. Over the 52 weeks, mdxRGT2 mice (47 ± 2 km; *p* < 0.05) ran more per week than mdxR (32 ± 2 km) and mdxRGT1 mice (Figure 5D; 31 ± 2 km). This was confirmed by relative analysis comparing weekly running distance to each mouse’s week 1 distance (Figure 5E). However, increased levels of voluntary wheel running in mdxRGT2 mice did not elevate their final treadmill time to fatigue over mdxRGT1 mice (Figure 5C).

### Microdystrophin gene therapy improved plantarflexor torque output, independent of construct or voluntary wheel running

**FIGURE 6:**
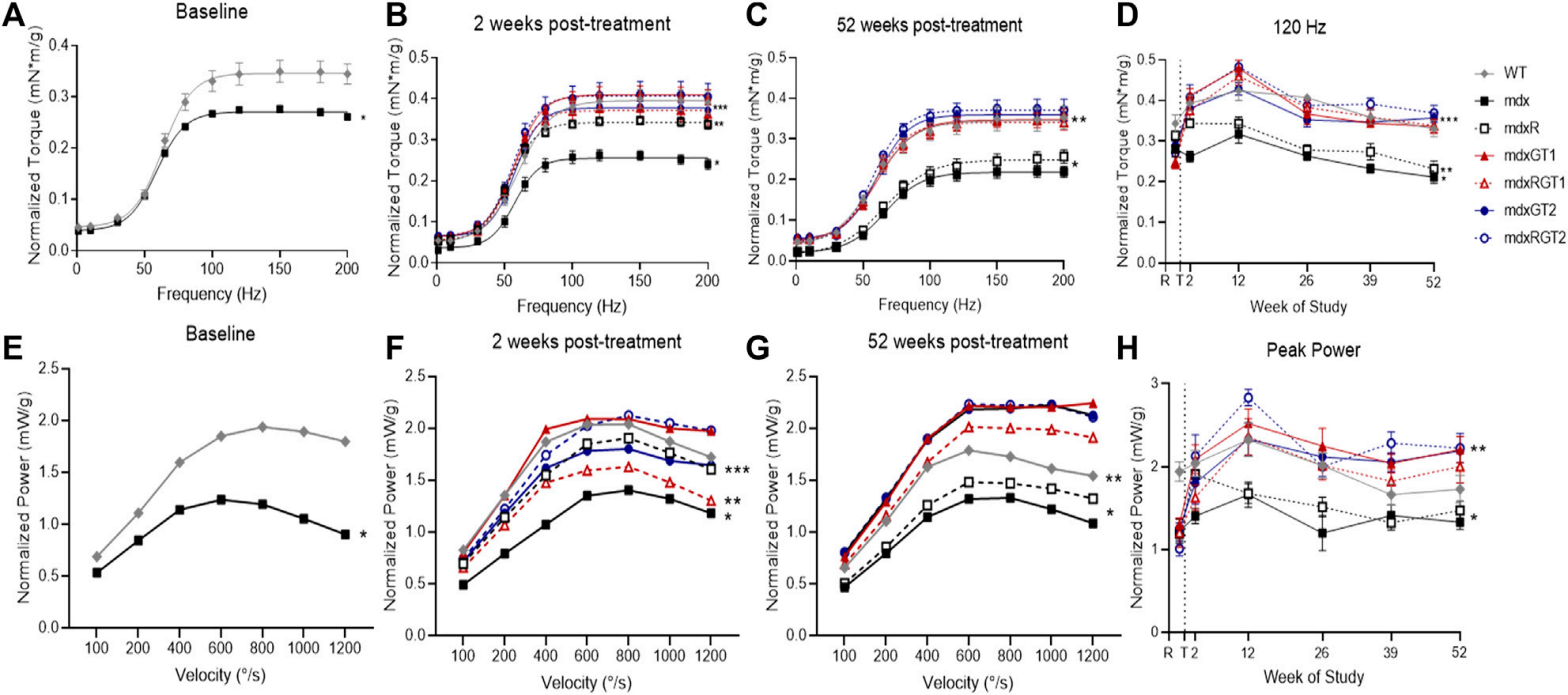
*In vivo* contractile properties of plantarillexors. Plantarflexor torque **(A–D)** and plantarflexor power **(E–H)**. **(A)** Baseline torque curve. *mdx < WT. **(B)** 2 weeks post-treatment. *mdx < all groups. **mdxR < mdxGT1, mdxRGT1, mdxRGT2. ***WT, mdxGT2, mdxRGT1 < mdxGT1, mdxRGT2. **(C)** 52 weeks post-treatment. *mdx, mdxR < all groups. **WT, mdxGT1, mdxRGT1<mdxRGT2. **(D)** Peak torque values (120 Hz) at all time points. *mdx < all groups. **mdxR < WT, mdxGT1, mdxRGT1, mdxGT2, mdxRGT2. ***mdxRGT2 > mdxGT2. **(E)** Baseline power curve. *mdx < WT. **(F)** 2 weeks post-treatment. *mdx < WT, mdxR, mdxGT1, mdxGT2, mdxRGT2. **mdxRGT1 < WT, mdxGT1, mdxRGT2. ***mdxGT2 < mdxGT1. **(G)** 52 weeks post-treatment. *mdx, mdxR < all other groups. **WT < mdxGT1, mdxGT2, mdxRGT2. **(H)** Peak power (800°/s) at all time points. *mdx, mdxR < all other groups. **mdxRGT2 > WT, mdxRGT1. All comparisons *p* < 0.05.

Contractile properties of plantar flexors were tested at baseline (1 week before treatment) and at 2-, 12-, 26-, 39-, and 52-weeks post-treatment to assess *in vivo* muscle function (Figure 6; Supplementary Table S6). Data are presented as mean ± SEM of normalized 120 Hz peak torque (mN*m/g) or 800°/s peak power (mW/g). At baseline, mdx mice (0.28 ± 0.02 mN*m/g) demonstrated low torque outputs compared to WT mice (0.34 ± 0.02 mN*m/g; *p* < 0.05) (Figure 6A). By 2 weeks, all groups treated with microdystrophin gene therapy with (mdxRGT1 0.38 ± 0.02 mN*m/g; mdxRGT2 0.41 ± 0.03 mN*m/g) and without (mdxGT1 0.41 ± 0.02 mN*m/g; mdxGT2 0.38 ± 0.01 mN*m/g) voluntary wheel running as well as untreated mice that performed voluntary wheel running (mdxR 0.35 ± 0.01 mN*m/g) improved torque output over mdx mice (0.26 ± 0.01 mN*m/g; all comparisons *p* < 0.05) (Figure 6B). By 12 weeks post-treatment, torque values of mdxR mice (0.34 ± 0.02 mN*m/g) returned to levels similar to mdx (0.32 ± 0.02 mN*m/g) (Figure 6D). At the end of the study, mice treated with both gene therapies, with (mdxRGT1 0.34 ± 0.02 mN*m/g; mdxRGT2 0.37 ± 0.02 mN*m/g) and without (mdxGT1 0.34 ± 0.02 mN*m/g; mdxGT2 0.36 ± 0.01 mN*m/g) voluntary wheel running, maintained their torque outputs similar to WT (0.33 ± 0.02 mN*m/g) and were greater than both untreated mdx groups (mdx 0.21 ± 0.02 mN*m/g; mdxR 0.23 ± 0.02 mN*m/g; *p* < 0.05). mdxRGT2 mice produced the highest torque values at the final time point (Figure 6C). Similarly, plantarflexor power of mdx mice (0.99 ± 0.09 mW/g) at baseline was lower than WT (1.55 ± 0.18 mW/g,; *p* < 0.05) (Figure 6E). However, by 52 weeks post-treatment, mdxGT1 (1.83 ± 0.22 mW/g), mdxGT2 (1.82 ± 0.21 mW/g), and mdxRGT2 (1.83 ± 0.21 mW/g) mice displayed higher power outputs vs. WT mice (1.44 ± 0.15 mW/g; *p* < 0.05) (Figure 6G). Post-treatment, mdx (1.40 ± 0.08 mW/g) and mdxR (1.58 ± 0.10 mW/g) mice produced lower power than all other groups across the course of the study (WT 1.95 ± 0.12 mW/g; mdxGT1 2.22 ± 0.08 mW/g; mdxRGT1 1.96 ± 0.12 mW/g; mdxGT2 2.10 ± 0.09 mW/g; mdxRGT2 2.29 ± 0.14 mW/g; all comparisons *p* < 0.05 (Figure 6H).

### Microdystrophin gene therapy two (GT2) better preserved diaphragm function when combined with prolonged endurance exercise

**FIGURE 7:**
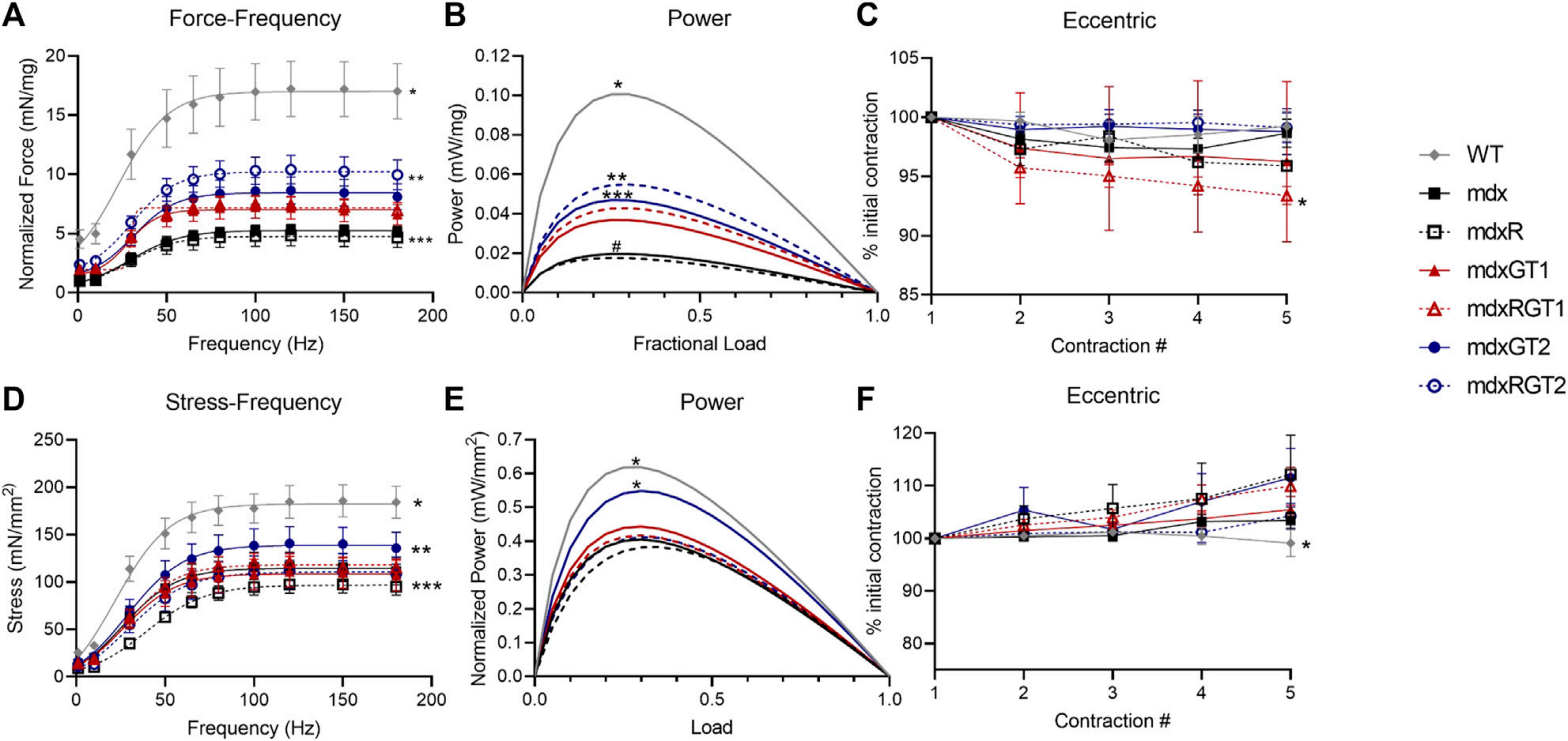
*Ex vivo* contractile properties. Diaphragm **(A–C)** and soleus **(D–F)**. **(A)** *WT > all other groups. **mdxRGT2 > mdxGT1, mdxRGT1, mdxGT2. ***mdx, mdxR < all other groups. **(B)** *WT > all other groups. **mdxRGT2 > mdxGT1, mdxRGT1. ***mdxGT2 > mdxGT1. ^#^mdx, mdxR < all other groups. **(C)** *mdxRGT1 <mdxRGT2. **(D)** *WT > all groups. **mdxGT2 > mdxR, mdxGT1, mdxRGT2. ***mdxR < mdxRGT1. **(E)** *WT, mdxGT2 > all other groups. **(F)** *WT < mdxR, mdxRGT1, mdxGT2. All comparisons *p* < 0.05.

At the conclusion of the study, mice were sacrificed, and *ex vivo* contractile experiments were conducted on diaphragm strips and whole soleus muscles (Figure 7; Supplementary Table S7). Absolute force and power, diaphragm strip mass, and soleus cross sectional area (CSA) are reported in Supplementary Table S7. Normalized forces and powers relative to strip mass for diaphragm and for soleus relative to CSA are described below to account for the potential bias of strip or muscle size. Diaphragm data are presented as mean ± SEM of normalized 120 Hz peak force (mN/mg) or power (mW/mg) at 40% maximum load (Figures 7A–C; Supplementary Table S7). WT mice produced the greatest diaphragm force (17.2 ± 2.3 mN/mg), while mdx (5.3 ± 0.4 mN/mg) and mdxR (4.8 ± 0.9 mN/mg) groups produced less force than all other groups (Figure 7A; *p* < 0.05). mdxRGT2 (10.1 ± 1.1 mN/mg; *p* < 0.05) produced higher force than other gene therapy-treated groups (mdxGT1 7.2 ± 1.0 mN/mg; mdxGT2 8.6 ± 1.1 mN/mg; mdxRGT1 7.7 ± 0.6 mN/mg). Similar to force, power analysis revealed that WT (0.095 ± 0.02 mW/mg) produced higher power than other groups, while mdx (0.019 ± 0.0 mW/mg) and mdxR (0.016 ± 0.0 mW/mg) values were depressed compared to all groups (Figure 7B; mdxGT1 0.035 ± 0.01 mW/mg; mdxRGT1 0.041 ± 0.01 mW/mg; mdxGT2 0.044 ± 0.01 mW/mg; mdxRGT2 0.052 ± 0.01 mW/mg; all comparisons *p* < 0.05). mdxRGT2 produced similar power to mdxGT2, and both produced higher power values than mdxGT1. mdxRGT2 also produced greater power than mdxRGT1 (*p* < 0.05). The only group that experienced minimal force loss (∼7%) due to eccentric contractions was mdxRGT1 (Figure 7C; *p* < 0.05).

### Soleus function demonstrated better force outputs in sedentary mice treated with microdystrophin gene therapy two (GT2)

Soleus data are presented as mean ± SEM of normalized 120 Hz peak stress (mN/mm^2^) or power (mW/mm^2^) at 40% maximum load (Figures 7D–F; Supplementary Table S7). The soleus stress-frequency measurements displayed a low force output in mdx groups (*p* < 0.05) compared to WT (184.8 ± 17.4 mN/mm^2^). mdxGT2 (140.8 ± 17.7 mN/mm^2^) mice displayed greater soleus stress over mdxR (97.4 ± 8.9 mN/mm^2^), mdxGT1 (110.9 ± 16.2 mN/mm^2^), mdxRGT2 (111.9 ± 18.0 mN/mm^2^) (Figure 7D; *p* < 0.05). Power values in mdxGT2 (0.53 ± 0.06 mW/mm^2^) mice were similar to WT (0.58 ± 0.05 mW/mm^2^) and higher (*p* < 0.05) than all other groups (Figure 7E; mdx 0.39 ± 0.06 mW/mm^2^; mdxR 0.38 ± 0.08 mW/mm^2^; mdxGT1 0.42 ± 0.08 mW/mm^2^; mdxRGT1 0.40 ± 0.04 mW/mm^2^; mdxRGT2 0.40 ± 0.07 mW/mm^2^). Soleus muscles did not experience force loss when subjected to the eccentric injury protocol (Figure 7F). mdxR, mdxRGT1, and mdxGT2 displayed slightly elevated force output compared to WT (*p* < 0.05).

### Oxidative red quadriceps fibers of mdxRGT1 mice displayed elevated mitochondrial respiration to WT levels. Mitochondrial respiration of diaphragm fibers of mdxGT2 mice were rescued to WT levels

**FIGURE 8:**
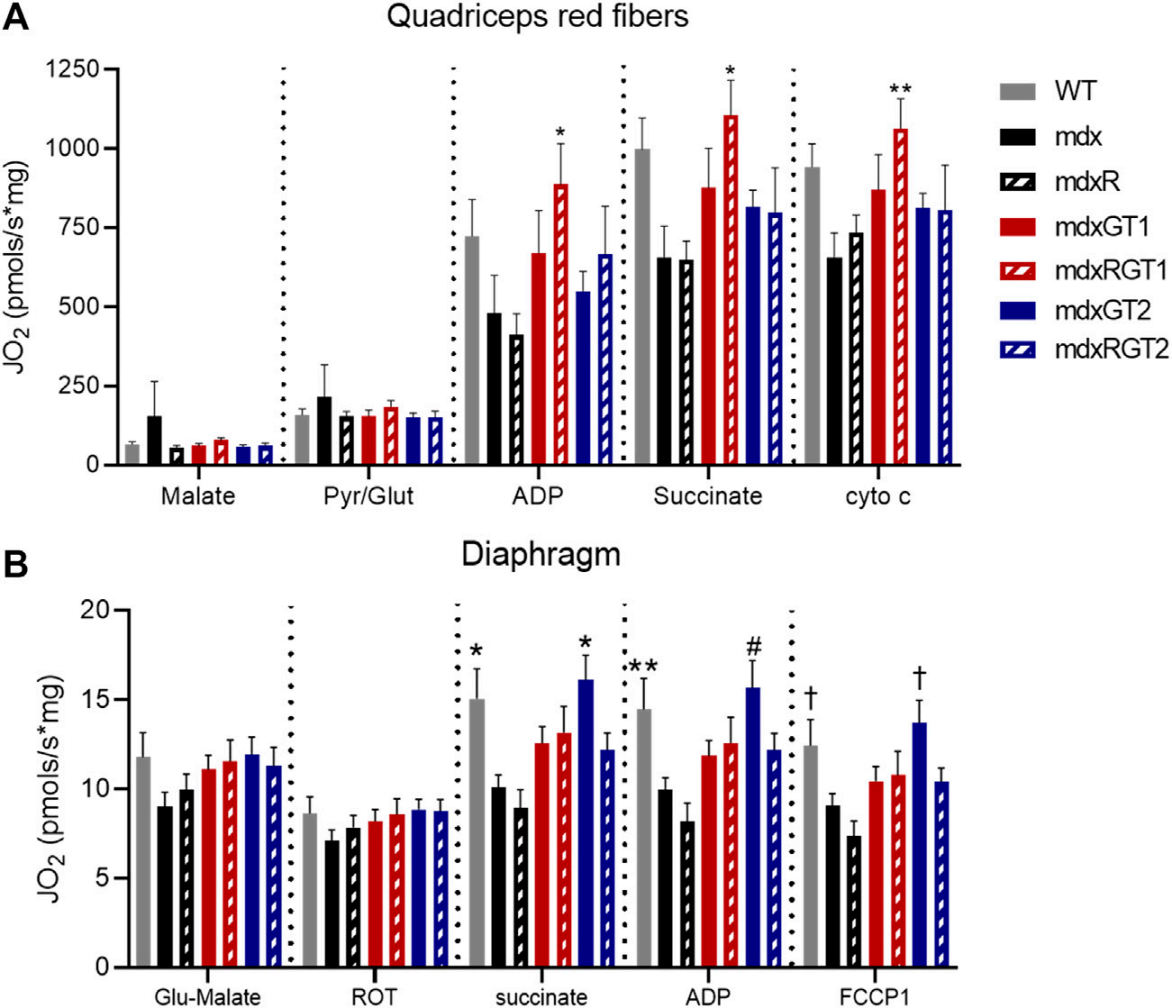
Mitochondrial respiration in red quadriceps and diaphragm fibers. **(A)** Red quadriceps. *mdxRGT1 > mdx, mdxR. **mdxRGT1 > mdx. **(B)** Diaphragm. *WT, mdxGT2 > mdx, mdxR. **WT > mdxR. ^#^mdxGT2 > mdx, mdxR. ^†^WT, mdxGT2 > mdxR. All comparisons *p* < 0.05.

Mitochondrial respiration, a key measure of oxidative capacity, was evaluated in red quadriceps fibers and diaphragm strips (Figure 8). Red muscle has a higher proportion of oxidative fibers. Data are presented as mean ± SEM pmols/s*mg. mdxRGT1 improved succinate-stimulated respiration of red quadriceps fibers almost two-fold over mdx mice (Figure 8A). ADP-stimulated respiration was similarly improved over both mdx and mdxR mice. In diaphragm, both WT (11.5 ± 8.3 pmols/s*mg) and mdxGT2 (11.1 ± 8.9 pmols/s*mg) mice had ∼30% greater succinate-stimulated respiration than mdx (8.8 ± 3.6 pmols/s*mg) and mdxR (5.2 ± 3.4 pmols/s*mg) groups (Figure 8B; *p* < 0.05). WT (11.4 ± 8.8 pmols/s*mg) increased ADP-stimulated respiration over mdxR (4.7 ± 3.0 pmols/s*mg), while mdxGT2 (11.0 ± 9.1 pmols/s*mg) respiration was higher than both mdx (8.7 ± 3.6 pmols/s*mg) and mdxR values; all comparisons *p* < 0.05.

### mdxRGT1 mice demonstrated the highest number of positive changes in metabolic enzymes of limb muscle compared to other groups

Metabolic enzyme activities were assessed in gastrocnemius (gastroc) and triceps (Supplementary Figures S2, 3). Citrate synthase (CS), malate dehydrogenase (MD) and cytochrome C oxidase (COX) activities were measured in both red and white gastrocnemius (Supplementary Figures S2A–C), while phosphofructokinase (PFK) and β-hydroxyacyl-CoA-dehydrogenase (βHAD) were only measured in red (Supplementary Figures S2D, E). Enzyme activities were higher in red vs. white muscle (Supplementary Figures S2A–C). CS activity in red gastroc was elevated in mdxRGT1 compared to mdx and WT groups (Supplementary Figures S2A). Both mdxRGT1 and mdxRGT2 displayed increased βHAD activity compared to mdx mice in red muscle (Supplementary Figures S2E). mdxRGT2 βHAD activity was also higher than WT. White muscle showed no differences between groups Supplementary Figures S2E; all comparisons *p* < 0.05.

In triceps, both treated running groups displayed elevated CS activity compared to mdx and mdxGT1 (Supplementary Figures S3A). In addition, mdxRGT2 CS activity was greater than WT, mdxR, and mdxGT2 (Supplementary Figures S3A). Interestingly, mdxR triceps had higher MD activity than mice treated with gene therapy alone, regardless of construct. mdxR COX activity was also higher than mdxGT1. Triceps PFK activity was greater in mdxRGT1 than WT and both sedentary treated groups. No differences in βHAD activity of triceps were observed Supplementary Figures S2E; all comparisons *p* < 0.05.

## Discussion

### Study objectives

The objectives of our current study were to determine if (1) prolonged (52 weeks) voluntary wheel running and (2) microdystrophin construct structure (± nNOS binding site) in mdx mice would sustain the functional benefits we reported for microdystrophin combined with short-term (21 weeks) voluntary wheel running in mdx mice (Hamm et al., 2021). We tested two microdystrophin constructs, μDys-5R (GT1), that includes the nNOS binding site and H2μDys (GT2), that does not include the nNOS binding site. We tested these two microdystrophin constructs with and without voluntary wheel running using comprehensive whole-body endurance and muscle function assessments in mdx mice. We performed these assessments on 7 groups: mdx, mdxR, mdxGT1, mdxRGT1, mdxGT2, mdxRGT2 and WT mice.

### Main findings

(1) Both microdystrophin constructs improved running capacity alone, but even moreso when combined with voluntary wheel running; (2) improvements in plantarflexor torque were comparable between all gene therapy treated groups, independent of running; (3) mdxRGT2 diaphragms performed better in *ex vivo* contractile assessments than other treated groups, with or without running; (4) mdxGT2 mice demonstrated higher force outputs in *ex vivo* soleus; (5) mdxRGT1 mice displayed the greatest adaptation to mitochondrial function in red quadriceps fibers as well as the highest number of adaptations in metabolic enzyme activity measured in limb muscles; and, (6) mdxGT2 mice produced diaphragm mitochondrial respiration rates similar to WT and higher than other mdx groups. Thus, prolonged voluntary wheel running complemented the functional improvements provided by both microdystrophin constructs but also revealed the unique benefits of each microdystrophin construct to dystrophic muscle. The experimental outcomes that distinguish the gene therapy-treated groups highlight the benefits of each construct and may help to elucidate their unique mechanisms of rescue.

### Microdystrophin as treatment for dystrophic muscle

DMD causes progressive skeletal muscle weakness, respiratory deficits, and cardiomyopathy. (Duan et al., 2021). Accordingly, mdx mice display decreased muscle strength and power, which is especially progressive in the diaphragm with age. (Dupont-Versteegden and McCarter, 1992; Lynch et al., 1997). In cardiac muscle, young mdx mice display electrocardiographic abnormalities but have very mild fibrosis or overall pathology. (Chu et al., 2002; Van Erp et al., 2010). Microdystrophin gene therapy is one strategy being used to restore muscle function to dystrophic muscle. Microdystrophin construct optimization has been a topic of interest in DMD research for some time. (Rafael et al., 1996; Chamberlain and Chamberlain, 2017; Duan, 2018a; Ramos et al., 2019). Several constructs are currently being tested in clinical trials, including constructs with and without the nNOS binding site (Chamberlain and Chamberlain, 2017; Duan, 2018a) that are similar to those used in the present study. With the potential success of clinical trials forthcoming, it is becoming increasingly important to determine the limitations of these microdystrophin constructs, especially in relation to exercise. At present, the combination of exercise and microdystrophin treatments are best assessed pre-clinically.

### Outcomes of uDys-5R (GT1) ± exercise

μDys-5R (GT1) is a newer microdystrophin construct that contains spectrin-like repeats 16 and 17, which form its unique nNOS-binding site. Unlike other microdystrophins, μDys-5R (GT1) can localize nNOS to the sarcolemma, like full-length dystrophin. (Lai et al., 2013). Dystrophic limb muscles experience ischemia during exercise. (Sander et al., 2000; Zhang et al., 2013). μDys-5R (GT1) has been shown to improve muscle perfusion in mdx mice during uphill treadmill exercise, likely due to its proper localization of nNOS. Our data demonstrated that GT1 improved running capacity alone, but even moreso when combined with voluntary wheel running to support the potential influence of the nNOS binding site. GT1 has also been shown to restore diaphragm force to WT levels and was protective during an eccentric injury protocol. (Lai et al., 2009). In the current study, we observed that mdx mice treated with GT1 displayed lower diaphragm force and power than WT mice and the activity-matched mdx mice treated with GT2. Conversely, mdxRGT1 mice improved mitochondrial respiration in red quadriceps, as well as CS activity in red gastroc. CS activity is a biomarker of mitochondrial density, so this combination indicates positive mitochondrial adaptations in red muscle to improve oxidative capacity. Both mdxRGT1 and mdxRGT2 displayed elevated βHAD activity compared to mdx, perhaps indicating a shift to favoring fatty acid oxidation to support endurance exercise. In the triceps, both treated groups that performed voluntary wheel running had elevated CS. Interestingly, triceps MD, and COX activity were upregulated in mdxR mice only. mdxRGT1 also displayed elevation of PFK enzymatic activity in triceps, indicating higher levels of glycolytic activity. Although all running groups experienced some metabolic changes compared to sedentary groups, mdxRGT1 displayed the greatest benefit. One potential explanation is that the nNOS-binding site improved blood flow to exercising limb muscles and in turn drove endurance-induced adaptations in mdxRGT1 mice. This potential mechanism should be explored in future studies. (Ferry et al., 2015).

### Outcomes of H2μDys (GT2) ± exercise

H2μDys (GT2) has been described as being less beneficial to muscle than variations with H3 and μDys-5R (GT1). (Lai et al., 2009; Duan, 2018b; Ramos et al., 2019). Nevertheless, we recently demonstrated that H2μDys gene therapy combined with 21 weeks of voluntary wheel running was beneficial to mdx mice. (Hamm et al., 2021). Treadmill endurance capacity of mdx mice was improved with gene therapy but improved even further with the addition of voluntary wheel running. (Hamm et al., 2021). Similarly, herein, our data demonstrated that GT2 improved running capacity alone, but similar to GT1 demonstrated increased running capacity with voluntary wheel running in mdxRGT2 mice similar to that of mdxRGT1 mice. Notably, this similar running capacity suggests the absence of the nNOS binding site in GT2 does not limit adaptation to running, and also suggests the possibility of other adaptive mechanisms to enhance endurance. A potential detriment of H2μDys (GT2) is the polyproline site that is thought to create chronic strain on the myotendinous junctions of the Achilles tendon, which anchors the soleus and gastrocnemius muscles to the calcaneus. (Banks et al., 2010). The soleus and gastrocnemius are the two main plantarflexors and are critical to the “push off” phase of gait during walking and running. Previous studies have shown improvements in plantarflexor and soleus function in mdx mice after voluntary wheel running. (Hayes and Williams, 1996; Baltgalvis et al., 2012; Selsby et al., 2013). We also reported improvements in plantarflexor torque were independent of running, but running was not detrimental. (Hamm et al., 2021). Herein, we demonstrated peak plantarflexor torque was improved with running (mdxR) over mdx mice across all time points post-treatment. Peak torque was further improved over untreated mice by both GT1 and GT2 constructs, regardless of activity level. mdxRGT2 mean peak plantarflexor torque was higher than mdxGT2 post-treatment, However, assessment of the plantarflexor soleus *ex vivo*, revealed force and power that were higher in sedentary mdxGT2 mice compared to other treated groups. These data suggest that voluntary wheel running may not be as beneficial to soleus muscle force and power of GT2 treated mice, but nevertheless the mdxRGT2 mice could run exceptionally well.

### Effects of running and the GT1 and GT2 microdystrophins on the cardiorespiratory system

#### Diaphragm

Respiratory failure and cardiomyopathy are two primary causes of mortality in patients with DMD. Herein, we focused on the functional improvements in the diaphragm. The diaphragm is critical to respiratory function and is one of the most affected muscles, recapitulating DMD pathology moreso than other muscles in mdx mice (Stedman et al., 1991; Dupont-Versteegden and McCarter, 1992; Lynch et al., 1997). Therefore, examining diaphragm performance is key to preclinical studies in mdx mice. It is unclear whether prolonged voluntary wheel running is beneficial (Dupont-Versteegden et al., 1994) or detrimental to diaphragm function of mdx mice. (Selsby et al., 2013). In the present study, diaphragm force was similarly low in both mdxR and mdx groups. Additionally, our data suggest that voluntary wheel running differentially affected the diaphragms of mice treated with each gene therapy. mdxRGT1 and mdxRGT2 mice each performed exceptionally well in treadmill tests, indicating a robust respiratory capacity. However, mitochondrial respiration of the running groups remained similar to mdx levels in the diaphragm, indicating that their exceptional endurance capacity is not due to diaphragm adaptations in mitochondrial respiration. Interestingly, mdxRGT2 mice demonstrated the best *ex vivo* diaphragm function of all mdx groups, while mdxRGT1 function was improved to a more moderate degree. Microdystrophin expression measured by immunofluorescence was lower in diaphragms of GT1-treated mice (mdxGT1, mdxRGT1) compared to GT2-treated mice (mdxGT2, mdxRGT2). In addition, diaphragm muscle pathology was slightly worse in mdxGT1 mice. Collectively, these data could explain the reduced diaphragm force outputs in mdx mice treated with GT1 (mdxGT1, mdxRGT1) herein. However, *ex vivo* diaphragm force was depressed in treated mice that ran compared with sedentary treated mice. (Hamm et al., 2021). In the current study, diaphragm force output was higher in mdxRGT2 mice compared with mdxGT2 mice. Based on these data, it appears that with age, voluntary wheel running can be beneficial to maintaining force output in the diaphragms of mice treated with GT2. Diaphragm force output was improved in both groups treated with GT1 (mdxGT1, mdxRGT1) but to a lesser degree compared to mdxRGT2 and WT. Therefore, running may not be as beneficial to GT1-treated diaphragms.

#### Cardiomyopathy

The cardiac phenotype of young mdx mice is mild. Several studies show that using a cardiac-specific AAV serotype, such as AAV9, induces robust microdystrophin expression, normalizes electrical activity, and prevents progression of pathophysiology in several mouse models of DMD. (Bostick et al., 2008; Shin et al., 2011; Howard et al., 2021). Previous studies in young, untreated mdx mice indicated that voluntary wheel running induces changes in the structure of the heart, some positive (Selsby et al., 2013) and some detrimental. (Costas et al., 2010; Hourdé et al., 2013). In our study, we did not directly assess cardiac function, but found that untreated mdx mice that performed voluntary wheel running (mdxR) showed a similarly mild cardiac dystrophic pathology compared to mdx mice. mdxR mice also performed as well as mdxGT2 mice, and even better than mdxGT1 mice in the treadmill test. Recently and herein we show that young mdx mice treated with GT2 gene therapy are not only able to tolerate exercise but thrive when performing endurance tests, especially after performing voluntary wheel running. (Hamm et al., 2021). In addition, after 52 weeks of running, at 14 months old, treated mice display low to no cardiac dystrophic pathology. Although assumptions about cardiac function cannot be fully extrapolated from these data, our findings suggest that either microdystrophin gene therapy combined with voluntary exercise is not detrimental to cardiac muscle of young mdx mice.

## Summary

The purpose of this study was to determine if voluntary wheel running would be beneficial to mdx mice treated with either the GT1 or the GT2 microdystrophin construct. Among the main findings, both microdystrophin constructs improved running capacity when combined with voluntary wheel running; while improvements in plantarflexor torque were comparable between all gene therapy treated groups, independent of running. Our data also indicated that the nNOS-binding site in GT1 may promote endurance exercise-driven adaptations in metabolic enzyme activity and the mitochondria of limb muscle. Further, in the diaphragm, GT2 can result in a high degree of force production when prolonged endurance exercise is applied but the addition of running to animals treated with this construct decreases mitochondrial respiration. These findings demonstrate that prolonged (52 weeks) voluntary wheel running and a microdystrophin construct structure with or without the nNOS binding site in mdx mice will sustain the functional benefits we reported for microdystrophin combined with short-term (21 weeks) voluntary wheel running in mdx mice (Hamm et al., 2021). Our findings further demonstrate that prolonged voluntary wheel running complemented the functional improvements provided by both microdystrophin constructs but also revealed the unique benefits of each to dystrophic muscle. Our data also suggest further studies should be conducted to evaluate the hardiness of each construct in different muscles and to elucidate the mechanisms that drive these differential adaptations.

## Data Availability

The raw data supporting the conclusions of this article will be made available by the authors, without undue reservation.

## References

[B1] Aartsma-Rus A. van Putten M. (2014). Assessing functional performance in the <em&gt;Mdx&lt;/em&gt; mouse model. J. Vis. Exp. 85, 51303. 10.3791/51303 PMC4158772 24747372

[B2] Baltgalvis K. A. Call J. A. Cochrane G. D. Laker R. C. Yan Z. Lowe D. A. (2012). Exercise training improves plantarflexor muscle function in mdx mice. Med. Sci. Sports Exerc 44, 1671–1679. 10.1249/MSS.0b013e31825703f0 22460476 PMC3470762

[B3] Banks G. B. Judge L. M. Allen J. M. Chamberlain J. S. (2010). The polyproline site in Hinge 2 influences the functional capacity of truncated dystrophins. PLoS Genet. 6, e1000958. 10.1371/journal.pgen.1000958 20502633 PMC2873924

[B4] Bostick B. Shin J. H. Yue Y. Duan D. (2011). AAV-microdystrophin therapy improves cardiac performance in aged female mdx mice. Mol. Ther. 19, 1826–1832. 10.1038/mt.2011.154 21811246 PMC3188746

[B5] Bostick B. Yue Y. Lai Y. Long C. Li D. Duan D. (2008). Adeno-associated virus serotype-9 microdystrophin gene therapy ameliorates electrocardiographic abnormalities in mdx mice. Hum. Gene Ther. 19, 851–856. 10.1089/hum.2008.058 18666839 PMC2888653

[B6] Chamberlain J. R. Chamberlain J. S. (2017). Progress toward gene therapy for duchenne muscular dystrophy. Mol. Ther. 25, 1125–1131. 10.1016/j.ymthe.2017.02.019 28416280 PMC5417844

[B7] Chu V. Otero J. M. Lopez O. Sullivan M. F. Morgan J. P. Amende I. (2002). Electrocardiographic findings in mdx mice: A cardiac phenotype of duchenne muscular dystrophy. Muscle Nerve 26, 513–519. 10.1002/mus.10223 12362417

[B8] Costas J. M. Nye D. J. Henley J. B. Plochocki J. H. (2010). Voluntary exercise induces structural remodeling in the hearts of dystrophin-deficient mice. Muscle Nerve 42, 881–885. 10.1002/mus.21783 21104863

[B9] Duan D. Goemans N. Takeda S. Mercuri E. Aartsma-Rus A. (2021). Duchenne muscular dystrophy. Nat. Rev. Dis. Prim. 7, 13–19. 10.1038/s41572-021-00248-3 33602943 PMC10557455

[B10] Duan D. (2018). Micro-dystrophin gene therapy goes systemic in duchenne muscular dystrophy patients. Hum. Gene Ther. 29, 733–736. 10.1089/hum.2018.012 29463117 PMC6066190

[B11] Duan D. (2018). Systemic AAV micro-dystrophin gene therapy for duchenne muscular dystrophy. Mol. Ther. 26, 2337–2356. 10.1016/j.ymthe.2018.07.011 30093306 PMC6171037

[B12] Dupont-Versteegden E. E. McCarter R. J. (1992). Differential expression of muscular dystrophy in diaphragm versus hindlimb muscles of mdx mice. Muscle Nerve 15, 1105–1110. 10.1002/mus.880151008 1406767

[B13] Dupont-Versteegden E. E. McCarter R. J. Katz M. S. (1994). Voluntary exercise decreases progression of muscular dystrophy in diaphragm of mdx mice. J. Appl. Physiol. 77, 1736–1741. 10.1152/jappl.1994.77.4.1736 7836193

[B14] Ferry A. Benchaouir R. Joanne P. Peat R. A. Mougenot N. Agbulut O. (2015). Effect of voluntary physical activity initiated at age 7 months on skeletal hindlimb and cardiac muscle function in mdx mice of both genders. Muscle and Nerve 52, 788–794. 10.1002/mus.24604 25704632

[B15] Gao Q. McNally E. M. (2015). The dystrophin complex: Structure, function and implications for therapy. Compr. Physiol. 5, 1223–1239. 10.1002/cphy.c140048 26140716 PMC4767260

[B16] Hamm S. E. Fathalikhani D. D. Bukovec K. E. Addington A. K. Zhang H. Perry J. B. (2021). Voluntary wheel running complements microdystrophin gene therapy to improve muscle function in mdx mice. Mol. Ther. - Methods and Clin. Dev. 21, 144–160. 10.1016/j.omtm.2021.02.024 33850950 PMC8020351

[B17] Harper S. Q. Hauser M. A. DelloRusso C. Duan D. Crawford R. W. Phelps S. F. (2002). Modular flexibility of dystrophin: Implications for gene therapy of duchenne muscular dystrophy. Nat. Med. 8, 253–261. 10.1038/nm0302-253 11875496

[B18] Hayes A. Williams D. A. (1996). Beneficial effects of voluntary wheel running on the properties of dystrophic mouse muscle. J. Appl. Physiol. 80, 670–679. 10.1152/jappl.1996.80.2.670 8929614

[B19] Hourdé C. Joanne P. Medja F. Mougenot N. Jacquet A. Mouisel E. (2013). Voluntary physical activity protects from susceptibility to skeletal muscle contraction-induced injury but worsens heart function in mdx mice. Am. J. Pathol. 182, 1509–1518. 10.1016/j.ajpath.2013.01.020 23465861

[B20] Howard Z. M. Dorn L. E. Lowe J. Gertzen M. D. Ciccone P. C. Rastogi N. (2021). Micro-dystrophin gene therapy prevents heart failure in an improved Duchenne muscular dystrophy cardiomyopathy mouse model. JCI Insight 6, e146511. 10.1172/jci.insight.146511 33651713 PMC8119181

[B21] Lai Y. Thomas G. D. Yue Y. Yang H. T. Li D. Long C. (2009). Dystrophins carrying spectrin-like repeats 16 and 17 anchor nNOS to the sarcolemma and enhance exercise performance in a mouse model of muscular dystrophy. J. Clin. Invest. 119, 624–635. 10.1172/JCI36612 19229108 PMC2648692

[B22] Lai Y. Zhao J. Yue Y. Duan D. (2013). α2 and α3 helices of dystrophin R16 and R17 frame a microdomain in the α1 helix of dystrophin R17 for neuronal NOS binding. Proc. Natl. Acad. Sci. U. S. A. 110, 525–530. 10.1073/pnas.1211431109 23185009 PMC3545791

[B23] Landisch R. M. Kosir A. M. Nelson S. A. Baltgalvis K. A. Lowe D. A. (2008). ADAPTIVE AND NONADAPTIVE RESPONSES TO VOLUNTARY WHEEL RUNNING BY mdx MICE. Muscle Nerve 38, 1290–1303. 10.1002/mus.21141 18816601 PMC3392332

[B24] Liu M. Yue Y. Harper S. Q. Grange R. W. Chamberlain J. S. Duan D. (2005). Adeno-Associated virus-mediated microdystrophin expression protects young mdx muscle from contraction-induced injury. Mol. Ther. 11, 245–256. 10.1016/j.ymthe.2004.09.013 15668136 PMC2581717

[B25] Lynch G. S. Rafael J. A. Hinkle R. T. Cole N. M. Chamberlain J. S. Faulkner J. A. (1997). Contractile properties of diaphragm muscle segments from old mdx and old transgenic mdx mice. Am. J. Physiol. 272, C2063–C2068. 10.1152/ajpcell.1997.272.6.C2063 9227435

[B26] Rafael J. A. Cox G. A. Corrado K. Jung D. Campbell K. P. Chamberlain J. S. (1996). Forced expression of dystrophin deletion constructs reveals structure-function correlations. J. Cell. Biol. 134, 93–102. 10.1083/jcb.134.1.93 8698825 PMC2120912

[B27] Ramos J. N. Hollinger K. Bengtsson N. E. Allen J. M. Hauschka S. D. Chamberlain J. S. (2019). Development of novel micro-dystrophins with enhanced functionality. Mol. Ther. 27, 623–635. 10.1016/j.ymthe.2019.01.002 30718090 PMC6403485

[B28] Rodgers B. D. Bishaw Y. Kagel D. Ramos J. N. Maricelli J. W. (2020). Micro-dystrophin gene therapy partially enhances exercise capacity in older adult mdx mice. Mol. Ther. - Methods and Clin. Dev. 17, 122–132. 10.1016/j.omtm.2019.11.015 31909085 PMC6939027

[B29] Sander M. Chavoshan B. Harris S. A. Iannaccone S. T. Stull J. T. Thomas G. D. (2000). Functional muscle ischemia in neuronal nitric oxide synthase-deficient skeletal muscle of children with Duchenne muscular dystrophy. Proc. Natl. Acad. Sci. U. S. A. 97, 13818–13823. 10.1073/pnas.250379497 11087833 PMC17659

[B30] Schill K. E. Altenberger A. R. Lowe J. Periasamy M. Villamena F. A. Rafael-Fortney J. A. (2016). Muscle damage, metabolism, and oxidative stress in mdx mice: Impact of aerobic running. Muscle Nerve 54, 110–117. 10.1002/mus.25015 26659868 PMC4905810

[B31] Selsby J. T. Acosta P. Sleeper M. M. Barton E. R. Sweeney H. L. (2013). Long-term wheel running compromises diaphragm function but improves cardiac and plantarflexor function in the mdx mouse. J. Appl. Physiol. (1985) 115, 660–666. 10.1152/japplphysiol.00252.2013 23823150 PMC3763072

[B32] Shin J. H. Nitahara-Kasahara Y. Hayashita-Kinoh H. Ohshima-Hosoyama S. Kinoshita K. Chiyo T. (2011). Improvement of cardiac fibrosis in dystrophic mice by rAAV9-mediated microdystrophin transduction. Gene Ther. 18, 910–919. 10.1038/gt.2011.36 21451578

[B33] Stedman H. H. Sweeney H. L. Shrager J. B. Maguire H. C. Panettieri R. A. Petrof B. (1991). The mdx mouse diaphragm reproduces the degenerative changes of Duchenne muscular dystrophy. Nature 352, 536–539. 10.1038/352536a0 1865908

[B34] Van Erp C. Loch D. Laws N. Trebbin A. Hoey A. J. (2010). Timeline of cardiac dystrophy in 3-18-month-old MDX mice. Muscle Nerve 42, 504–513. 10.1002/mus.21716 20589894

[B35] Yiu E. M. Kornberg A. J. (2015). Duchenne muscular dystrophy. J. Paediatr. Child. Health 51, 759–764. 10.1111/jpc.12868 25752877

[B36] Zhang Y. Yue Y. Li L. Hakim C. H. Zhang K. Thomas G. D. (2013). Dual AAV therapy ameliorates exercise-induced muscle injury and functional ischemia in murine models of Duchenne muscular dystrophy. Hum. Mol. Genet. 22, 3720–3729. 10.1093/hmg/ddt224 23681067 PMC3749861

