# Supplementary material for: Prolonged voluntary wheel running reveals unique adaptations in mdx mice treated with microdystrophin constructs ± the nNOS-binding site
Source: Front Physiol. 2023 Jun 26;14:1166206. doi: 10.3389/fphys.2023.1166206 (PMC10330712; doi:10.3389/fphys.2023.1166206)
Supplement: Supplementary file 1 [file DataSheet1.pdf]

Supplemental Figures

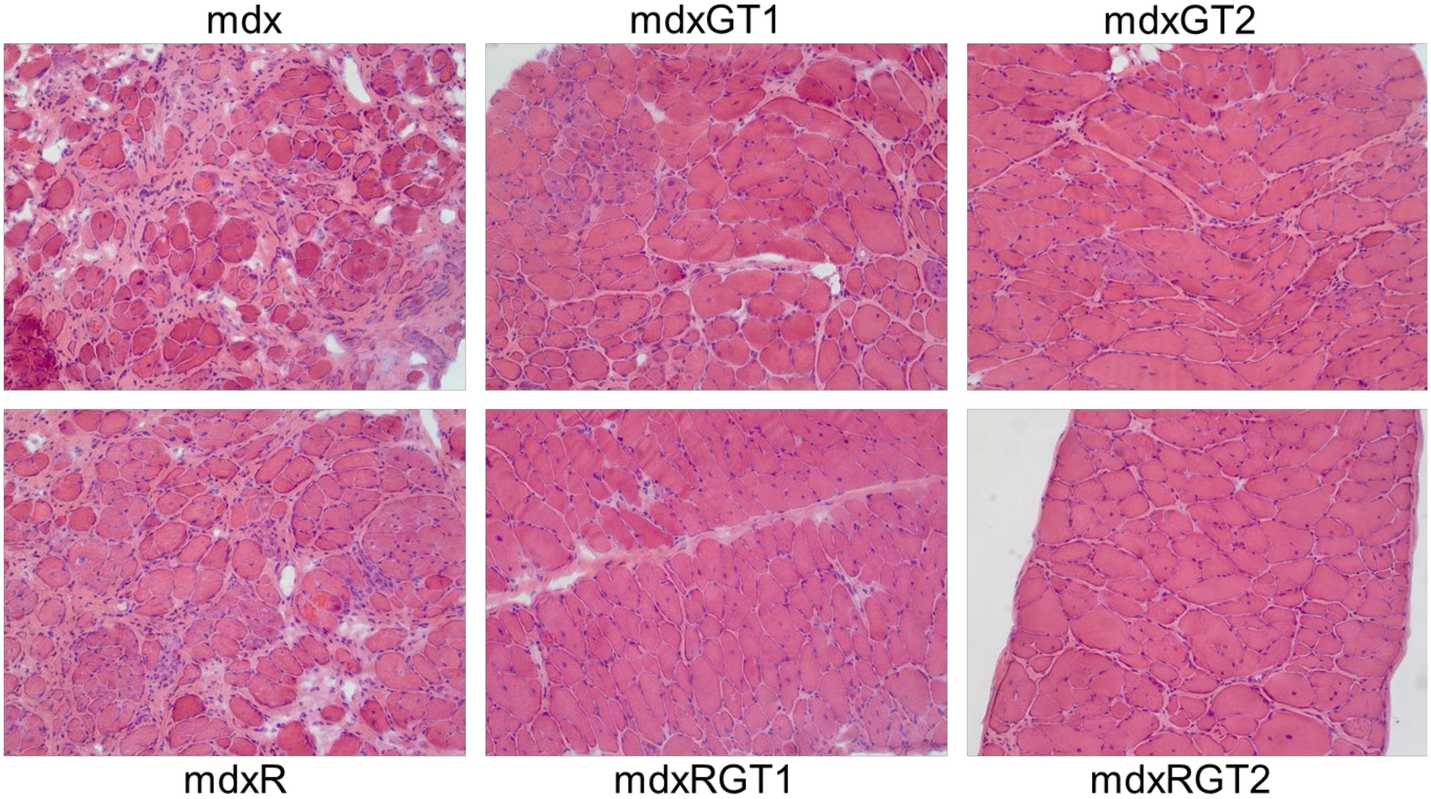

Figure S1. Representative images of diaphragm stained with hematoxylin and eosin, showing histopathology of mdx groups. Data was quantified as dystrophic grade (**Fig. 4**). mdx and mdxR show greater active dystrophic pathology compared to treated groups.

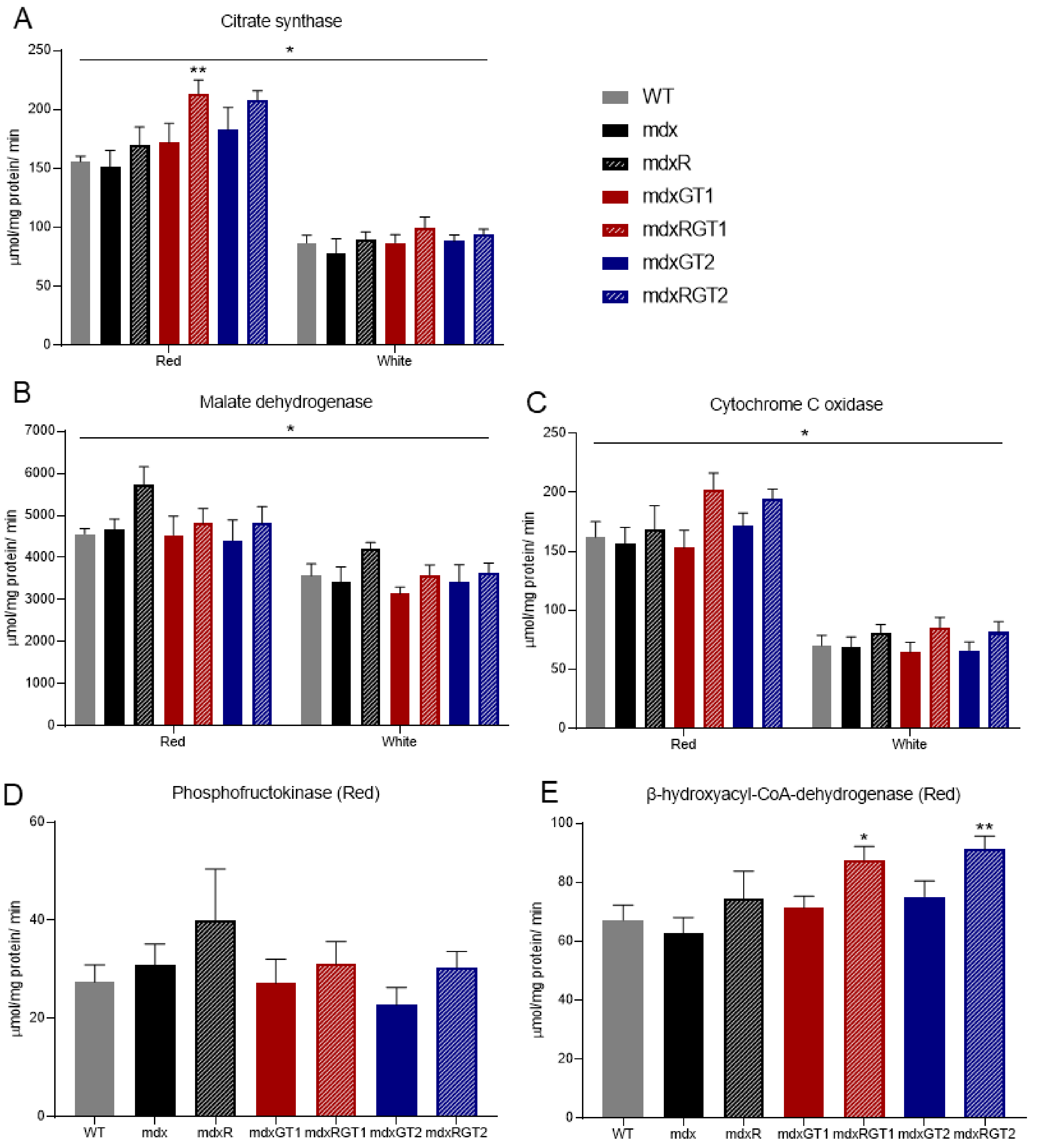

A
Citrate synthase
B
Malate dehydrogenase
C
Cytochrome C oxidase
D
Phosphofructokinase (Red)
E
β-hydroxyacyl-CoA-dehydrogenase (Red)
μmol/mg protein/ min
Red
White
WT
mdx
mdxR
mdxGT1
mdxRGT1
mdxGT2
mdxRGT2
*
**

Figure S2. Metabolic enzyme activity in red (A-E) and white gastrocnemius (A-C). (**A**) Citrate synthase activity, *red > white. **mdxRGT1 > mdx, WT. (**B**) Malate dehydrogenase activity, *red > white. (**C**) Cytochrome C oxidase activity, *red > white. (**D**) Phosphofructokinase activity, No differences. (**E**) β-hydroxyacyl-CoA-dehydrogenase activity, *mdxRGT1 > mdx. **mdxRGT2 > mdx, WT. All comparisons $p<0.05$.

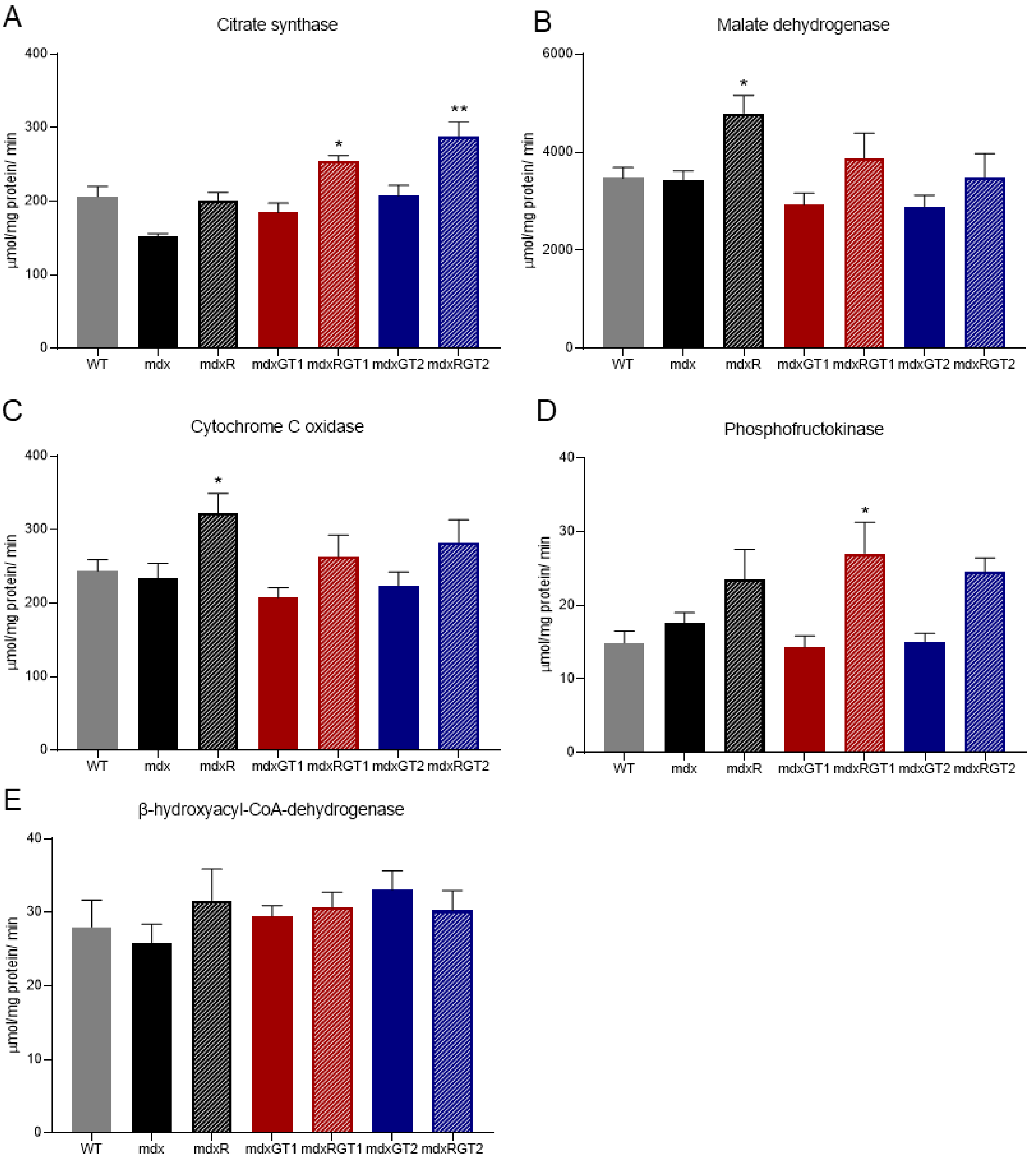
A
Citrate synthase
µmol/mg protein/ min
0
100
200
300
400
*
**
WT
mdx
mdxR
mdxGT1
mdxRGT1
mdxGT2
mdxRGT2
B
Malate dehydrogenase
µmol/mg protein/ min
0
2000
4000
6000
*
WT
mdx
mdxR
mdxGT1
mdxRGT1
mdxGT2
mdxRGT2
C
Cytochrome C oxidase
µmol/mg protein/ min
0
100
200
300
400
*
WT
mdx
mdxR
mdxGT1
mdxRGT1
mdxGT2
mdxRGT2
D
Phosphofructokinase
µmol/mg protein/ min
0
10
20
30
40
*
WT
mdx
mdxR
mdxGT1
mdxRGT1
mdxGT2
mdxRGT2
E
β-hydroxyacyl-CoA-dehydrogenase
µmol/mg protein/ min
0
10
20
30
40
WT
mdx
mdxR
mdxGT1
mdxRGT1
mdxGT2
mdxRGT2

Figure S3. Metabolic enzyme activity in triceps. (**A**) Citrate synthase activity, *mdxRGT1 > mdx, mdxGT1. **mdxRGT2 > WT, mdx, mdxR, mdxGT1, mdxGT2. (**B**) Malate dehydrogenase activity, *mdxR > mdxGT1, mdxGT2. (**C**) Cytochrome C oxidase activity, *mdxR > mdxGT1. (**D**) Phosphofructokinase activity, *mdxRGT1 > WT, mdxGT1, mdxGT2. (**E**) β-hydroxyacyl-CoA-dehydrogenase activity, No differences. All comparisons $p<0.05$.

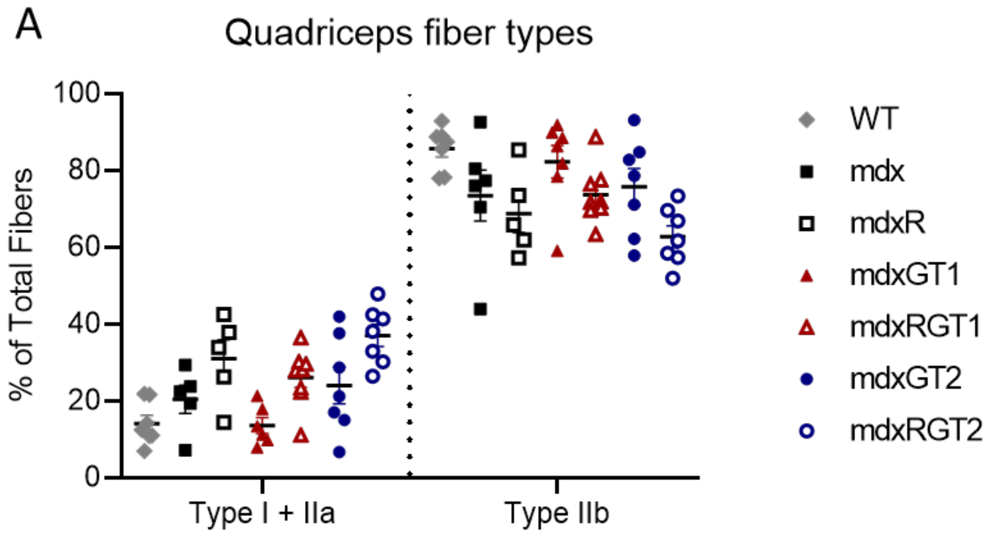

Figure S4. Fiber type proportion in quadriceps. Type I and IIa grouped together to represent oxidative fibers. (**A**) Proportion of fiber type, no differences. All comparisons $p<0.05$.

Supplemental Tables

Table S1. Western blot quantification of microdystrophin protein in diaphragm, quadriceps, and heart muscles, normalized to standard microdystrophin reference sample; Mean % ± SE.

	**WT**	**mdx**	**mdxR**	**mdxGT1**	**mdxRGT1**	**mdxGT2**	**mdxRGT2**
Diaphragm	2±0	3±0	2±0	68±11	60±12	73±4	74±10
Quadriceps	1±0	1±0	2±0	64±5	62±6	53±4	48±4
Heart	10±3	10±3	9±6	184±17	176±17	194±18	214±12

Table S2. Microdystrophin (mdxGT1, mdxRGT1, mdxGT2, mdxRGT2) and dystrophin (WT) quantification of DYSB expression in diaphragm, quadriceps, and heart muscle fibers as percent positivity. Mean % ± SE.

	**WT**	**mdx**	**mdxR**	**mdxGT1**	**mdxRGT1**	**mdxGT2**	**mdxRGT2**
Diaphragm	98±1	0±0	0±0	56±7	66±8	89±3	90±3
Quadriceps	100±0	1±0	1±0	87±5	83±3	91±4	86±3
Heart	100±0	0±0	0±0	100±0	100±0	100±0	99±1

Table S3. Dystrophic grade in diaphragm, heart, and quadriceps muscles. Mean ± SE.

	**WT**	**mdx**	**mdxR**	**mdxGT1**	**mdxRGT1**	**mdxGT2**	**mdxRGT2**
Diaphragm	0.0±0.0	3.9±0.1	3.6±0.4	2.7±0.2	2.5±0.2	1.7±0.2	1.6±0.3
Heart	0.0±0.0	1.0±0.4	1.2±0.5	0.0±0.0	0.3±0.3	0.0±0.0	0.0±0.0
Quadriceps	0.0±0.0	2.0±0.3	1.8±0.3	1.4±0.1	1.4±0.1	1.4±0.1	1.6±0.2

Table S4. Treadmill time to fatigue; Mean minutes ± SE.

	**WT**	**mdx**	**mdxR**	**mdxGT1**	**mdxRGT1**	**mdxGT2**	**mdxRGT2**
Baseline (min)	91±12	54±10	35±13	52±13	45±7	35±8	48±8
3 wk post (min)	79±7	29±6	38±15	60±5	95±7	80±7	81±14
13 wk post (min)	83±8	29±8	81±17	62±6	122±4	77±6	116±5
27 wk post (min)	83±6	50±9	107±9	77±4	130±2	91±8	133±4
40 wk post (min)	78±10	46±9	115±4	74±7	134±6	89±11	136±7
53 wk post (min)	81±11	33±7	108±5	75±8	125±4	96±11	141±7

Table S5. Running wheel activity; Mean ± SE. Distance in km/week: Total distance in kilometers (km)/52 weeks; Running wheel activity: Percent of mean distance over 52 weeks/week 1 distance. *mdxRGT2 > mdxR, mdxRGT; $p<0.05$.

	**mdxR**	**mdxRGT1**	**mdxRGT2**
Running wheel distance (km/week)	32±2	31±2	47±2*
Running wheel activity (% of week 1)	87±5	82±5	157±9*

Table S6. *In vivo* plantarflexor torque at 120 Hz and power data at 800°/s presented as mean ± SE.

	**WT**	**mdx**	**mdxR**	**mdxGT1**	**mdxRGT1**	**mdxGT2**	**mdxRGT2**
Baseline 120 Hz (mN*m/g)	0.34±0.02	0.28±0.02	0.31±0.01	0.24±0.01	0.25±0.02	0.29±0.02	0.27±0.01
2 wk post 120 Hz (mN*m/g)	0.39±0.02	0.26±0.01	0.35±0.01	0.41±0.02	0.38±0.02	0.38±0.01	0.42±0.03
12 wk post 120 Hz (mN*m/g)	0.43±0.03	0.32±0.02	0.34±0.02	0.48±0.02	0.46±0.02	0.43±0.02	0.48±0.02
26 wk post 120 Hz (mN*m/g)	0.41±0.01	0.26±0.01	0.28±0.01	0.37±0.03	0.38±0.02	0.35±0.02	0.39±0.01
39 wk post 120 Hz (mN*m/g)	0.36±0.02	0.23±0.01	0.27±0.02	0.35±0.01	0.36±0.02	0.35±0.01	0.39±0.02
52 wk post 120 (mN*m/g)	0.33±0.02	0.21±0.02	0.23±0.02	0.34±0.02	0.34±0.02	0.36±0.01	0.37±0.02
Baseline 800°/s (mW/g)	1.9±0.1	1.2±0.1	1.2±0.1	1.3±0.1	1.2±0.2	1.3±0.1	1.0±0.1
2 wk post 800°/s (mW/g)	2.0±0.2	1.4±0.1	1.9±0.1	2.0±0.2	1.6±0.2	1.8±0.3	2.1±0.3
12 wk post 800°/s (mW/g)	2.3±0.2	1.7±0.1	1.7±0.1	2.5±0.2	2.4±0.2	2.3±0.2	2.8±0.1
26 wk post 800°/s (mW/g)	2.0±0.2	1.2±0.2	1.5±0.1	2.2±0.2	2.0±0.2	2.1±0.1	2.0±0.1
39 wk post 800°/s (mW/g)	1.7±0.1	1.4±0.1	1.3±0.1	2.0±0.1	1.8±0.1	2.1±0.1	2.3±0.1
52 wk post 800°/s (mW/g)	1.7±0.2	1.3±0.1	1.5±0.1	2.2±0.2	2.0±0.2	2.2±0.1	2.2±0.2

Table S7. *Ex vivo* diaphragm and soleus (SOL) muscle morphology and contractile data; Mean ± SE. Mean forces are peak force measured at 120 Hz stimulation frequency. Mean power is estimated peak power measured at 40% of maximal load.

	**WT**	**mdx**	**mdxR**	**mdxGT1**	**mdxRGT1**	**mdxGT2**	**mdxRGT2**
Diaphragm mass (mg)	8.6±0.8	10.2±1.0	10.7±2.0	10.5±1.0	11.2±0.8	13.1±0.6	11.6±0.9
SOL CSA (mm^2)	1.0±0.1	1.3±0.1	2.2±0.0	1.4±0.1	1.7±0.1	1.4±0.1	1.5±0.1

	WT	mdx	mdxR	mdxGT1	mdxRGT1	mdxGT2	mdxRGT2
Diaphragm 120 Hz absolute force (mN)	142.2±16.9	55.1±8.3	54.0±16.2	74.3±10.4	84.5±7.1	116.3±17.4	118.4±17.3
Diaphragm 120 Hz force (mN/mg)	17.3±2.3	5.3±0.4	4.8±0.9	7.2±1.0	7.7±0.6	8.6±1.1	10.1±1.1
SOL 120 Hz absolute force (mN)	184.7±12.0	143.0±15.1	223.2±16.9	146.9±20.4	212.1±22.0	186.9±21.5	162.9±23.3
SOL 120 Hz stress (mN/ mm^2)	184.8±17.4	116.8±14.9	100.1±7.5	110.9±16.2	121.2±9.7	140.8±17.7	111.9±18.0
Diaphragm 40% absolute power (mW)	0.77±0.13	0.20±0.05	0.19±0.07	0.37±0.05	0.45±0.05	0.59±0.08	0.69±0.13
Diaphragm 40% power (mW/mg)	0.095±0.02	0.019±0.0	0.016±0.0	0.035±0.01	0.041±0.01	0.044±0.01	0.052±0.01
SOL 40% absolute power (mW)	0.59±0.05	0.48±0.07	0.84±0.18	0.56±0.11	0.69±0.07	0.70±0.07	0.58±0.09
SOL 40% power (mW/ mm^2)	0.58±0.05	0.39±0.06	0.38±0.08	0.42±0.08	0.40±0.04	0.53±0.06	0.40±0.07

Table S8. Sample size for each assessment. Note O2k assays were run in duplicate or triplicate when feasible.

	WT n=	mdx n=	mdxR n=	mdxGT1 n=	mdxRGT1 n=	mdxGT2 n=	mdxRGT2 n=
**Western blot**							
Diaphragm	7	6	5	7	8	7	7
Quadriceps	7	6	5	7	8	7	7
Heart	7	6	5	7	8	7	7
**IF**							
Diaphragm	7	5	4	7	8	7	7
Quadriceps	7	5	5	7	8	7	7
Heart	7	5	5	7	8	7	7
**Histopathology**							
Diaphragm	7	6	5	7	8	7	7
Quadriceps	7	6	5	7	8	7	7
Heart	7	6	5	7	8	7	7
**Treadmill**							
Baseline	7	6	5	7	8	7	7
3 wk post	7	6	5	7	8	7	7
13 wk post	7	6	5	7	8	7	7
27 wk post	7	6	5	7	8	7	7
40 wk post	7	6	5	7	8	7	7
53 wk post	7	6	5	7	8	7	7
**Torque**							
Baseline	7	7	8	8	8	8	8

	WT n=	mdx n=	mdxR n=	mdxGT1 n=	mdxRGT1 n=	mdxGT2 n=	mdxRGT2 n=
2 wk post	8	7	7	8	8	7	8
12 wk post	8	6	7	6	8	7	8
26 wk post	8	7	7	7	8	7	8
39 wk post	8	7	7	7	8	7	8
52 wk post	7	6	4	7	8	6	7
**Power**							
Baseline	8	7	7	7	7	6	8
2 wk post	7	7	7	8	8	7	8
12 wk post	8	7	7	7	8	7	7
26 wk post	8	7	7	6	8	7	8
39 wk post	8	7	7	7	8	7	8
52 wk post	6	6	5	7	7	7	7
**Ex vivo diaphragm**							
Force	5	7	5	7	8	7	7
Power	6	6	5	7	8	7	7
Eccentric	7	7	4	6	8	7	7
**Ex vivo soleus**							
Stress	7	7	5	7	8	7	7
Power	7	7	5	7	8	7	7
Eccentric	7	7	5	6	8	6	6
**O2k**							
Red quad	14	14	10	14	16	14	14
Diaphragm	17	16	11	17	19	14	16
**Metabolic enzymes**							
CS, red G	7	6	5	7	8	7	7
CS, white G	7	5	5	7	8	7	7
MD, red G	7	6	5	7	8	7	7
MD, white G	7	6	5	7	8	7	7
Cyto C, red G	7	6	5	7	8	7	7
Cyto C, white G	7	6	5	7	8	7	7
PFK, red G	7	6	5	7	8	7	7
BHAD, red G	7	6	5	7	8	7	7
CS, tri	7	6	5	7	8	7	7
MD, tri	7	6	5	7	8	7	7
Cyto C, tri	7	6	5	7	8	7	7
PFK, tri	7	6	5	7	8	7	7
BHAD, tri	7	6	5	7	8	7	7
**Fiber types (quad)**							
Type I + IIa	7	5	5	6	8	7	7
Type IIb	7	6	5	7	8	7	7

Table S9. Mouse mass at sacrifice by group; Mean ± SE.

	WT	mdx	mdxR	mdxGT1	mdxRGT1	mdxGT2	mdxRGT2
Mouse mass (g)	38.2±2.2	38.3±2.5	34.7±0.7	35.9±0.8	36.1±0.9	38.6±1.3	34.6±0.7
